# Bayesian species delimitation in *Pleophylla* chafers (Coleoptera) – the importance of prior choice and morphology

**DOI:** 10.1186/s12862-016-0659-3

**Published:** 2016-05-05

**Authors:** Jonas Eberle, Rachel C. M. Warnock, Dirk Ahrens

**Affiliations:** Zoologisches Forschungsmuseum Alexander Koenig Bonn, Centre of Taxonomy and Evolutionary Research, Adenauerallee 160, 53113 Bonn, Germany; Department of Entomology, Natural History Museum, London, SW7 5BD UK; Department of Life Sciences, Silwood Park Campus, Imperial College London, Ascot, SL7 5PY UK; School of Earth Sciences, University of Bristol, Bristol, BS8 1RJ UK

**Keywords:** Scarabaeidae, Aedeagus, Eigenshape analysis, Speciation, Phylomorphospace, Integrative taxonomy, Bayesian species delimitation

## Abstract

**Background:**

Defining species units can be challenging, especially during the earliest stages of speciation, when phylogenetic inference and delimitation methods may be compromised by incomplete lineage sorting (ILS) or secondary gene flow. Integrative approaches to taxonomy, which combine molecular and morphological evidence, have the potential to be valuable in such cases. In this study we investigated the South African scarab beetle genus *Pleophylla* using data collected from 110 individuals of eight putative morphospecies. The dataset included four molecular markers (*cox1*, 16S, *rrnL*, ITS1) and morphometric data based on male genital morphology. We applied a suite of molecular and morphological approaches to species delimitation, and implemented a novel Bayesian approach in the software iBPP, which enables continuous morphological trait and molecular data to be combined.

**Results:**

Traditional morphology-based species assignments were supported quantitatively by morphometric analyses of the male genitalia (eigenshape analysis, CVA, LDA). While the ITS1-based delineation was also broadly congruent with the morphospecies, the *cox1* data resulted in over-splitting (GMYC modelling, haplotype networks, PTP, ABGD). In the most extreme case morphospecies shared identical haplotypes, which may be attributable to ILS based on statistical tests performed using the software JML. We found the strongest support for putative morphospecies based on phylogenetic evidence using the combined approach implemented in iBPP. However, support for putative species was sensitive to the use of alternative guide trees and alternative combinations of priors on the population size (*θ*) and rootage (*τ*_*0*_) parameters, especially when the analysis was based on molecular or morphological data alone.

**Conclusions:**

We demonstrate that continuous morphological trait data can be extremely valuable in assessing competing hypotheses to species delimitation. In particular, we show that the inclusion of morphological data in an integrative Bayesian framework can improve the resolution of inferred species units. However, we also demonstrate that this approach is extremely sensitive to guide tree and prior parameter choice. These parameters should be chosen with caution – if possible – based on independent empirical evidence, or careful sensitivity analyses should be performed to assess the robustness of results. Young species provide exemplars for investigating the mechanisms of speciation and for assessing the performance of tools used to delimit species on the basis of molecular and/or morphological evidence.

**Electronic supplementary material:**

The online version of this article (doi:10.1186/s12862-016-0659-3) contains supplementary material, which is available to authorized users.

## Background

The identification and delimitation of species is one of the most crucial exercises in the assessment of biodiversity and in understanding the Tree of Life, because species occupy a central role in nearly all disciplines of biology. Species delimitation therefore has broad implications, from biological and ecological conservation, to comparative evolutionary analyses [1–4]. Despite the challenge and importance of defining species units, methods for delimiting species using independent sources of data (e.g., DNA and phenetic data) have only recently been proposed (e.g., [5–15]). Nevertheless, at least since Sneath and Sokal [16], there has been an extensive use of quantitative methods to infer similarity based on morphological traits. Broadly defined as “numerical taxonomy”, or phenetics, these methods have traditionally been used (and criticized) for inferring phylogenetic relationships (e.g., [17, 18]). However, integrative approaches to taxonomy shed new light on the utility of these methods, which have the potential to offer an independent, more reproducible way of inferring species limits [19].

In addition to controversy over the application of different species concepts and their impact for delimiting species [20], delimitation is expected to be especially challenging during the earliest stages of divergence, or speciation, when both molecular and morphological characters exhibit low levels of differentiation [21]. At this stage it can be extremely difficult to detect genetic isolation (i.e., the ultimate outcome of speciation) due to gene flow among populations and incomplete lineage sorting between species [22, 23]. Although molecular data can be useful for the rapid identification and delimitation of species, these processes can compromise the interpretation of the results. Incomplete lineage sorting – shared ancestral polymorphisms between species – can lead to perceived genetic similarity among phenotypically divergent species. Consequently, gene flow and incomplete lineage sorting can result in similar patterns among inferred gene trees [24–26]. To further complicate matters, introgressive hybridization – secondary gene flow between species – can also produce similar patterns among inferred gene trees (e.g., [27–29]).

A suite of new methods have been proposed that can incorporate incomplete lineage sorting in a multilocus framework for the estimation of species trees [30–33] and/or species delimitation [20, 33, 34]. Although these methods rely on the *a priori* assignment of individuals to pre-defined units (species or populations; [20]), they can be used to test explicit hypotheses of species delimitations. However, studies of recent radiations, or speciation in a young species, will be characterized by uncertain species designations, and are likely to remain challenging.

In contrast to DNA-based taxonomy, common practise for the traditional taxonomic treatment of taxa is an assessment of the organism’s entire morphology. In most groups of insects this includes detailed examination of the copulation organs, which often undergo rapid morphological divergence, driven by sexual selection [35]. However, quantitative data on insect genitalia are rarely obtained for the purposes of integrative taxonomy, and so methods for combining this type of morphological information with molecular data are still underdeveloped [19]. Previously, the only available methods for delimiting species on the basis of morphology were clustering approaches [8, 9, 36, 37]. Unfortunately, these methods quickly loose power when too many species are included, or when dealing with specimens whose closest phylogenetic relatives are unknown [7, 14]. Here we use morphometric and molecular data in an integrative framework, to delimit species in the scarab beetle genus *Pleophylla* Erichson, 1847. Following the recommendation of Carstens et al. [6], we implemented a suite of methods, including a recently developed approach that incorporates continuous morphological trait data with the multispecies coalescent [14, 34].

*Pleophylla* is a highly conspicuous genus, found only in isolated parts of the South African escarpment and the East African highlands. The genus belongs to the tribe Sericini (Coleoptera: Scarabaeidae), a highly diverse clade of herbivorous beetles with nearly 4,000 described species. The adults feed polyphagously on a variety of angiosperms, while the larva feed on humus and plant roots in the upper soil layers. Morphological and molecular evidence has shown that the genus belongs to one of the most ancestral-branching lineages of the Sericini, together with its presumptive sister group, *Omaloplia*, in the eastern Mediterranean [38, 39]. Members of the genus exhibit extreme homogeneity in external morphology, and identification of species usually relies on examination of the male genitalia – a trait used to commonly distinguish between homogenous species of insects [40], including most members of the tribe Sericini [41]. Current taxonomic classification recognises only three valid species ([42]; globalspecies.org/ntaxa/2359831; accessed Dec 13, 2015), however, an extensive survey and taxonomic revision of museum collections has identified 24 distinct morphospecies [43] (Eberle J, Beckett M, Özguel-Siemund A, Frings J, Fabrizi S, Ahrens D. Afromontane forests hide nineteen new species of ancient *Pleophylla *chafers (Coleoptera: Scarabaeidae): phylogeny and taxonomic revision, in preparation). The aim of our study was to provide a primer for the clarification of the taxonomy of this group, and to explore power and limitations of morphological, molecular and combined approaches to species delimitation in an integrative framework for an apparent “complex” case study.

## Methods

### Taxon sampling and molecular data collection

**Fig. 1:**
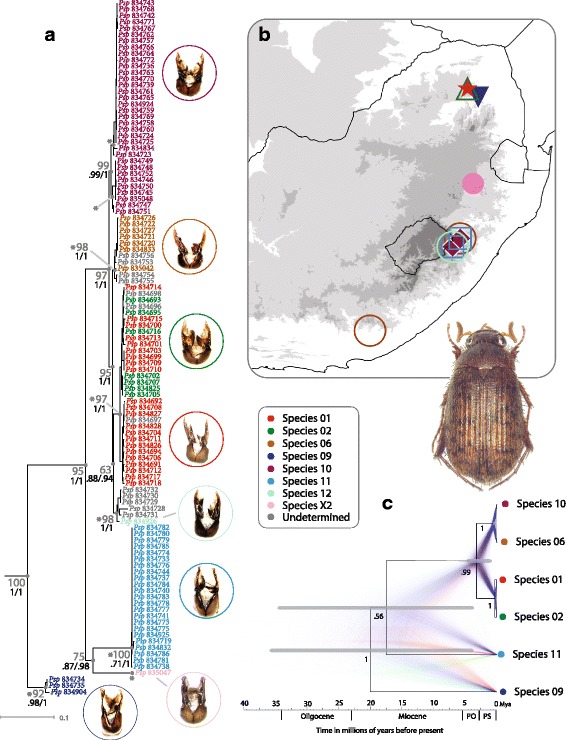
**a** Maximum likelihood (RAxML) tree of *Pleophylla* for the combined molecular dataset. Specimens are colored according to morphospecies (Additional file 1: Table S1). Branch length corresponds substitutions per site. Support values for ML and Bayesian posterior probabilities are shown next to branches in grey (RAxML) or indicated below (PhyML/MrBayes). ITS1 GMYC clusters are indicated by an asterisk (*). **b** Map of South African sampling localities (Additional file 1: Table S2). **c** Bayesian species tree obtained using *BEAST. Clade posterior probabilities are indicated next to branches. Confidence intervals (*grey bars*) show the upper limits of the 95 % HPDs obtained using a divergence rate for *cox1* of 2 % My-1, and the lower limits obtained using a rate of 4 %. Mean node ages arbitrarily correspond to the mean estimates obtained using a rate of 2 % My-1 (Additional file 1: Table S6). A cloudogram of 10,000 posterior samples shows the uncertainty in the inferred species tree, obtained using the program DensiTree [65]; different colours (*blue, red, green*) correspond to each consensus topology in the total set of trees

A total of 110 individuals of eight putative morphospecies of the genus *Pleophylla* were collected from eight localities in South Africa (Additional file 1: Table S1-S2; Fig. 1). So far, all known species are endemic to South Africa and represent a limited selection of the morphological diversity of *Pleophylla* (Eberle J, Beckett M, Özguel-Siemund A, Frings J, Fabrizi S, Ahrens D. Afromontane forests hide nineteen new species of ancient *Pleophylla *chafers (Coleoptera: Scarabaeidae): phylogeny and taxonomic revision, in preparation). Four of these species have not been described yet, therefore we refer to all putative morphospecies using the same numerical format throughout the text for consistency. *Omaloplia nigromarginata* and *O. ruricola* from the putative sister lineage of *Pleophylla* [38] were included as outgroup taxa. We assessed support for the monophyly of putative morphospecies using standard molecular markers – the nuclear ribosomal rRNA 28S gene, the nuclear internal transcribed spacer 1 (ITS1), and the mitochondrial cytochrome oxidase subunit 1 (*cox1*) and 16S rRNA (*rrnL*) genes. Details of DNA extraction, sequencing, alignment and model selection are provided in the Additional file 1.

### Morphometric analysis

The partial outline of the male’s left paramere (part of the intromittent genital organs, in dorsal view) (Additional file 1: Figure S1) was digitized from images captured on a microscope. The partial outline was extracted from 68 male specimens where the paramere was well preserved. The outlines were resampled as a set of 150 semi-landmarks using tpsDig 2.1 [44]. Standard eigenshape analysis [45, 46] was performed in Eigenshape 2.6, as implemented in morpho-tools [47]. Of the 67 eigenshape axes produced, further analysis was performed on the four eigenshape axes that together explained 75 % of the variation in the samples. Based on these informative eigenshape axes we performed a canonical variate analysis (CVA), grouping the samples according to the morphospecies assignments.

Model-based hierarchical clustering [37, 48] was applied to identify groups of individuals that resemble each other, independent of other evidence or *a priori* assignments, using the R package mclust 4.4 [36, 37]. The function *mclust* was used to evaluate the fit of all available clustering models to the morphometric data that explained 75 % (eigenshape axes 1–4) and 95 % (eigenshape axes 1–14) of total paramere shape variance. This method uses expectation maximization (EM) to estimate the maximum likelihood of alternative multivariate mixture models that describe shape variation in the morphometric data [49, 50], and estimates the optimal number of clusters based on the Bayesian Information Criterion (BIC) [51]. All models were evaluated for a predefined number of 1 to 20 clusters and the best-fit result was used for further analyses.

To assess the fit of the *a priori* morphospecies assignments and the hierarchical clusters found using mclust to the data, we performed a linear discriminant analysis based on the respective specimen groupings and calculated the probability of group membership for each individual. This was done using the R package MASS 7.3.35 [52]. The prior probability that a specimen belonged to a given group was set to be equal for all individuals and groups.

Finally, to investigate the impact of phylogeny on the inferred morphospace, the RAxML tree topology (based on the partitioned combined molecular dataset) was projected onto the paramere morphospace (eigenshape axes 1 and 2) using the function *phylomorphospace* in the R package phytools [53]. This function estimates the positions of the ancestral nodes using a maximum likelihood approach [53]. In addition, a three-dimensional version of this plot was produced based on eigenshape axes 1, 2 and 3 using the function *phylomorphospace3d*. The code was modified to make coloration for species group affiliation possible.

### Phylogenetic analysis

Phylogenetic analyses of individual and combined markers were performed using likelihood and Bayesian methods. Each analysis was run with the substitution model and partitions selected using PartitionFinder [54] (Additional file 1: Table S3). Unpartitioned maximum likelihood analysis was performed using PhyML 3.0 [55], and partitioned maximum likelihood analysis was performed using RAxML 7.3 [56, 57]. Bayesian phylogenetic analysis was performed using MrBayes 3.1.2 [58]. The default prior on branch lengths implemented in MrBayes can sometimes lead to spuriously large estimates of internal branch lengths [59, 60]. Because the GMYC approach to species delineation is sensitive to estimates of branch lengths, we ran four sets of analyses using an exponential prior on the branch lengths with mean = 0.1 (default), 0.05, 0.01 or 0.005 substitutions/site.

### Bayesian species tree estimation

The multispecies coalescent was implemented in *BEAST 1.75 [31, 61, 62] to co-estimate the species tree, individual (*cox1* and ITS1) gene trees and divergence times. The less informative ribosomal markers were excluded because analysis in *BEAST that included *rrnL* and 28S failed to converge, despite extensive efforts to improve convergence diagnostics. Putative morphospecies were used to define taxonomic units *a priori* – all 14 female individuals, for which there was ambiguity regarding *a priori* species assignment, were excluded from the analyses (Additional file 1: Table S1). The mean substitution rate of *cox1* was fixed, clock model parameters were unlinked across genes, and the rate of ITS1 was estimated relative to *cox1*. Estimates for the substitution rate of *cox1* among insect species vary substantially across different studies, and are dependent on a large number of variables [63, 64]. We therefore applied a range of mean branch rates, in five independent sets of analyses (2, 2.5, 3, 3.5 or 4 % My^−1^). The resulting posterior sample of species trees was additionally visualized with DensiTree [65]. Further details of all phylogenetic analyses, including prior parameter and chain settings, are provided in the Additional file 1.

### Distinguishing incomplete lineage sorting from hybridization

To assess whether low genetic variation observed among morphospecies could be attributed to incomplete lineage sorting, we used the posterior predictive checking approach developed by Joly et al. [66] and implemented in the software JML [67]. This approach uses simulated datasets of gene trees and sequence alignments generated under a coalescent model that assumes no migration (or hybridisation) for a given species tree. The proportion of simulated datasets for which the minimum pairwise distance is lower than the observed, can be interpreted as the posterior probability (*P*) that the model is correct. A small *P* value therefore suggests that a model that assumes no hybridization does not fit the data well (e.g., the observed minimum genetic distances are lower than expected). To account for uncertainty, simulations were performed for individual partitions using 10,000 trees from the posterior distribution of species tree output by *BEAST, which include estimates of population size and branch lengths. Further details of the simulations are provided in the Additional file 1.

### DNA-based species delimitation

For single marker species delimitation (*cox1* and ITS1) we used four widely implemented approaches: statistical parsimony analysis [68], automated barcode gap detection (ABGD) [69], the generalized mixed Yule-coalescent (GMYC) model [12, 70, 71], and the Poisson tree processes (PTP) model [72]. Outgroup species (*Omaloplia*) and specimens with duplicate haplotypes were pruned from the dataset (or tree) prior to analysis, otherwise some methods have been shown to produce false positives [73].

Haplotype networks for each individual marker were generated using statistical parsimony analysis [68] implemented in TCS 1.2 [42]. Statistical parsimony analysis partitions the data into networks of closely related haplotypes connected by changes that are non-homoplastic with a 95 % probability; if applied to mtDNA, the inferred networks have been found to be largely congruent with Linnaean species [74]. The GMYC model [12, 70, 71] was used to estimate species boundaries with the trees obtained from MrBayes and RAxML using in the R package *splits* [70], with single and multiple threshold options. This method is based on the phylogenetic species concept and identifies species clusters by recognising the apparent increase in the branching rate from interspecific diversification to population-level coalescence, and defining the threshold based on an ultrametric tree. Trees were converted to ultrametric using PATHd8 [75] and the penalized likelihood method implemented in r8s 1.7 [76], with the optimal smoothing parameter selected using the cross-validation procedure. The age of the ingroup was assigned an arbitrary age of 1, and the resultant trees were fully resolved using TreeEdit 1.0 [77] using an arbitrary branch length of 4 x 10^−6^. Finally, we estimated uncertainty in the number of GMYC species clusters based on the Akaike Information Criterion (AIC), using the method outlined in [78]. This approach uses a modified AIC score, corrected for sample size (AIC_c_), to assess the relative support for alternative (single and multiple threshold) models, versus the maximum likelihood model, and the null model (no change in the branching rate). Akaike weights (the relative support for each model) are assigned to each model based on the AIC_c_ scores. Model-averaged estimates of the number of GMYC species are obtained from the models withinδAIC_c_ = 2. The phylogenetic species concept also underlies the Poisson tree processes (PTP) model for species delimitation [72]. However, in contrast to the GMYC approach, the PTP infers speciation events based on a shift in the number of substitutions at internal nodes. We employed the maximum likelihood variant of PTP using the RAxML trees. For the ABGD approach we used the online version (last modified on 10/29/2015 and accessed on 01/23/2016, http://wwwabi.snv.jussieu.fr/public/abgd/abgdweb.html, [69]). This method is based on the assumption that divergence among organisms belonging to the same species will be less than the divergence observed among organisms of different species. The first significant gap in the distribution of sequence distances beyond intraspecific sequence divergence can thus be used to infer operational taxonomic units (OTU) that may be related to species (e.g., [79]). ABGD analyses were performed on matrices of pairwise sequence divergence, calculated for each marker using MEGA (v6.06, [80]). Distances were corrected using the best fitting substitution models. Prior maximum divergence of intraspecific diversity was set to 0.01, which has previously been demonstrated to recover species accurately [69].

Finally, the results of competing approaches to species delimitation were compared using the “entities counts” (i.e., inferred species counts) and the match ratio = 2*N_match_/(N_i_ + N_morph_), where N_match_ is the number of species with exact matches (i.e., all specimens of a given morphospecies – and only those – belong to a single GMYC entity) and N_i_ and N_morph_ are the number of inferred molecular operational taxonomic units (MOTUs) and morphospecies, respectively [73]. If there is complete congruence between the MOTU entities and the morphospecies the match ratio = 1, otherwise the ratio will be < 1.

### Total-evidence species delimitation

We assessed support for the *a priori* morphospecies assignments using a total-evidence-based Bayesian approach, implemented in the programs iBPP 2.1.2 [14] and BPP 3.0, [20, 34]. Briefly, this method uses a multispecies coalescent model to assess competing hypotheses of species delimitations, allowing for conflict between gene and species trees. The results are conditioned on a user specified guide tree and depend on estimates of the species divergence times (*τ*) and population sizes (*θ*). Individuals are assigned to independent populations and alternative delimitation hypotheses are proposed by collapsing one or more internals nodes in the guide tree. In the original implementation, the likelihood calculation is based on molecular data [34], while iBPP includes an extension of the model that allows continuous trait data to be included in the likelihood calculation [14]. This latter approach therefore enables both molecular and morphological data to be combined in the assessment of *a priori* species assignments.

It has been demonstrated that the results of this method can be sensitive to both prior parameter and guide tree choice [81]. For example, for high values of *θ* the model tends to (over-) split species, and for low values of *θ* the model tends to lump species together. To assess the robustness of our results, we compared the results obtained under variable combinations of the specified priors on the root age (*τ*_*0*_) and the population mutation rate (*θ*) (Table 1). To assess the influence of the guide tree, we compared the results obtained using three alternative input trees: (a) the topology estimated using *BEAST, (b) the topology estimated from the concatenated DNA matrix using RAxML/MrBayes, (c) a modified version of the *BEAST topology based on morphological similarity among species (Additional file 1: Figure S2). All combinations of prior parameter (Table 1) and guide tree choices were performed in iBPP (a) without data, to evaluate the impact of the priors, and using the following three datasets: (b) molecular data only, (c) morphometric data only, and (d) molecular and morphometric data. The analysis sometimes got stuck in a single species model, resulting in poor overall convergence, and so all analyses were repeated 10 times with different random seeds to ensure stability of the results.

**Table 1:** α and ß parameters, describing the prior distributions of the population mutation rate (θ) and the root age (τ_0_) parameters that were combined in the iBPP analyses

Prior	α	ß	Mean	Variation
*θ*_1_	1	10	0.1	0.01
*θ*_2_	1	20	0.05	2.5e-3
*θ*_3_	2	2000	0.001	5e-7
τ_0–1_	1	10	0.1	0.01
τ_0–2_	1	20	0.05	2.5e-3
τ_0–3_	2	2000	0.001	5e-7

In an additional set of analyses, we implemented unguided species delimitation using the program BPP [20]. This method accounts for uncertainty in the guide tree, by proposing changes to the species tree topology using nearest-neighbour interchange (NNI), as well as proposing changes to species assignments. Morphometric data cannot be analysed in BPP, so this analysis was performed for the molecular dataset only. The analyses were performed using the above combinations of priors and initial guide tree choices.

To explore the impact of distinct single-marker genotypes within the same morphospecies, in combination with the morphological trait data, we also analysed an additional guide tree with guided and unguided BPP, in which *sp10* was specified as two species entities (This split received strong support in several single marker delimitations, see results).

## Results

### Phylogenetic analysis and the monophyly of morphospecies

Phylogenetic analysis of independent and combined datasets using different approaches and parameter choices (PhyML, RAxML, and MrBayes) produced overall similar topologies (Fig. 1, Additional file 1: Figures S3-S5). Changing the branch length prior implemented in MrBayes had no impact on the inferred topology but had a large impact on tree length (the sum of branch lengths) (Additional file 1: Table S4). Analysis of different datasets (mitochondrial, nuclear or combined) mainly differed in their degree of tree resolution, and the level of support for the monophyly of individual morphospecies and/or interspecific relationships. There is remarkably low interspecific molecular variation observed across the entire genus. The trees produced using the ribosomal markers (*rrnL* and 28S) were poorly resolved. The *cox1* data provided better resolution and supported the monophyly of two out of eight putative morphospecies. ITS1 provided the best resolution and supported the monophyly of all but two morphospecies (*sp01* and *sp02*) (Additional file 1: Figure S3-S5).

**Fig. 2:**
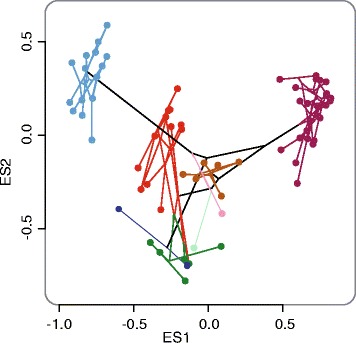
Plot of the 2D phylomorphospace using the RAxML tree topology (based on the partitioned combined molecular dataset) projected onto the paramere morphospace explained by eigenshape axes 1 and 2. Colors refer to Fig. 1

The topology obtained using the combined dataset that included all four markers was identical to the ITS1 gene tree (Fig. 1), but support values for most nodes were greater than those obtained using individual genes. In the combined analyses of all four markers, the monophyly of all putative morphospecies was strongly supported with the aforementioned exception. Morphospecies *sp01* and *sp02* were never recovered as monophyletic, although these groups occupied distinct areas of the morphospace in the morphometric analysis of the genitalia (Fig. 2, Additional file 1: Figure S1, Additional file 2).

The Bayesian species tree estimated using *BEAST for the combined *cox1* and ITS1 dataset resulted in strong support for the interspecific relationships estimated using the *cox1* data, rather than the ITS1 data. Although the species tree topology differed to that obtained using alternative phylogenetic methods (PhyML, RAxML and MrBayes), the individual gene trees (for *cox1* and ITS1) obtained using *BEAST were not different. The age of the most recent common ancestor of the sampled members of the genus, was estimated to be 2.64 – 35.97 and 3.69 – 17.88 Mya, based on the 95 % highest posterior density intervals for the slowest and fastest *cox1* substitution rates (2 and 4 % My^−1^), respectively (Additional file 1: Table S5). The use of a higher *cox1* substitution rate produced younger and, unexpectedly, more precise posterior age estimates. The ages for the two youngest divergence events (*sp01* + *sp02* and *sp06* + *sp10*) were estimated to be no older than 0.17 Mya and 0.65 Mya, respectively (Additional file 1: Table S6).

Evidence of hybridization was assessed using the posterior predictive checking approach as implemented in the software JML (Joly, 2012), based on the minimum pairwise sequence distances among morphospecies for each marker partition (*cox1* [P1 vs. P2 vs. P3], and ITS1), and the resulting posterior probability (*P*) of observing these distances under the multispecies coalescent model assuming no hybridization (Additional file 1: Table S7). In all cases the observed pairwise distances between individuals of all morphospecies were not lower than expected at the 5 % level (*P* > 0.05), given the null model (the coalescent with no migration or hybridization) across all partitions (*cox1* P1 and P2, *P* > 0.1; P3, *P* > 0.05; ITS1, *P* > 0.2). The distances observed between individuals of the two species pairs that could not be resolved using *cox1* (*sp06 + sp10*) or both *cox1* and ITS1 (*sp01 + sp02*) were not lower than expected for either marker (i.e., *sp06 + sp10*, *P* > 0.2; *sp01 + sp02*, *P >* 0.6). The tests performed suggest that incomplete lineage sorting is sufficient to explain the observed genetic variation (although mitochondrial partition P3 produced anomalous results for *sp09* and *sp11*, see Additional file 1: Table S7).

### Molecular tree- and character-based species delimitation

We investigated DNA-based species delimitation and associated uncertainty using (i) statistical parsimony, (ii) the GMYC model, (iii) the PTP model, and (iv) ABGD approach. The analyses using the *rrnL* and 28S data did not provide support for any of the putative morphospecies (results not shown). Of the 13 resulting *cox1* networks, three matched exclusively a single putative morphospecies. ITS1 networks provided a closer correspondence to the morphospecies. Of the 9 ITS1 networks, four matched exclusively a single putative morphospecies: *sp09*, *sp11*, *sp12* and *spX2*. Individuals of morphospecies *sp06* shared two networks, and individuals of morphospecies *sp10* shared two networks. Individuals of morphospecies *sp01* and *sp02* shared a single network. Together these results suggest that there is a higher degree of incomplete lineage sorting among *cox1* than ITS1, and that species *sp01 and sp02* cannot be distinguished on the basis of the molecular markers used here.

The GMYC results obtained using *cox1* were very sensitive to the input tree, but there were no obvious differences in the GMYC output that could be attributable to the trees generated using MrBayes versus RAxML, or PATHd8 versus r8s (Additional file 1: Table S8). Bayesian trees with longer branch lengths tended to result in more GMYC entities (species clusters + singletons), but not ubiquitously. Consequently, the *cox1* trees produced very variable results. In most cases several (up to 8) models contributed to a majority of the Akaike weight (>0.5), suggesting that no single model best represented the data. Accounting for uncertainty in model selection resulted in the number of entities ranging between 3.00 (σ^2^ = 0) and 16.54 (σ^2^ = 0.89), depending on the input tree; these GMYC units were widely incongruent with the *a priori* morphospecies assignments (further details therefore not shown here). There was less variation in the GMYC results obtained using the ITS1 trees – the single threshold models were always preferred to the multiple threshold models. In the majority of cases only one single threshold model was found within δAIC_c_ = 2, suggesting that the preferred model provided an appreciably better fit to the data than the alternatives. The ITS1 data resulted in a minimum of 8 (σ^2^ = 0) and a maximum of 10.99 (σ^2^ = 4.05) entities, depending on the input tree. In 8 out of 10 cases, the preferred model resulted in eight entities, corresponding to morphospecies *sp01* + *sp02*, *sp06*, *sp09*, *sp11*, *sp12*, *spX2*, and two clusters of morphospecies *sp10*.

In general, congruence between the inferred MOTUs and the morphospecies was more dependent on marker choice than species delimitation method (Table 2, Additional file 2: Table S9). For *cox1* the number of MOTUs ranged from 7 (PTP) to 13 (GMYC), while the analyses based on ITS1 resulted in 8 (GMYC, PTP, ABGD), 9 (TCS) and 10 (GMYC) entities. The PTP and ABGD analyses largely confirmed the results of the GMYC model for the ITS1 data; five of the eight MOTUs were fully congruent with the morphospecies (*sp11*, *spx2*, *sp9*, *sp6*, *sp12*). Finally, the match ratios obtained for *cox1* were consistently lower (0.27-0.42) than those obtained using ITS1 (0.47-0.63) (Table 2).

**Table 2:** DNA based species delimitation results

	*cox1*				ITS1			
	PTP	GMYC	TCS	ABGD	PTP	GMYC	TCS	ABGD
Entities	7	13	13^a^	11	8	8	9	8
Match ratio	0.27	0.29	0.30	0.42	0.63	0.63	0.47	0.63

The number of delimited entities and the match ratio (2*N_match_/(N_GMYC_ + N_morph_)) [73] after removing undetermined specimens is given

^a^contained one MOTU composed of only female specimens, this unit was not considered for match ratio estimation

### Morphometric evidence for species delimitation

We first assessed quantitative support for the eight putative morphospecies assignments among *Pleophylla* based on an open shape outline of the left paramere of the male genitalia, using (i) standard eigenshape analysis, (ii) canonical variate analysis (CVA), (iii) hierarchical clustering, and (iv) linear discriminant analysis. The first four eigenshape axes represented 75 % of the cumulative variation of the outline shape (Additional file 1: Table S10, Figure S6). Eigenaxis 1, 2, 3 and 4 represented 51.5 %, 15.6 %, 6.8 % and 6.0 % of the variation, respectively. The first 14 eigenshape axes account for 95 % of the cumulative variation. The plots of the 2D and 3D phylomorphospace (Fig. 2, Additional file 5) showed clear separation between all but one of the morphospecies, with no intermediate states between the morphospecies. The only exception wa*s sp12*, which overlapped in morphospace with *sp02*. CVA on eigenshape axes 1–4 (Additional file 1: Figure S1) revealed a clear distinction between five of the eight morphospecies (*sp01*, *sp02*, *sp06*, *sp10* and *sp11*), with the exception of those for which only one or two specimens were available for analysis (*sp09*, *sp12* and *spX2*). This was in contrast to the DNA-based tree topology and species delimitation, where specimens of two species pairs (*sp01 + sp02*, and *sp06* + *sp10*) could not be distinguished based on the analysis of *cox1* and/or ITS1.

**Fig. 3:**
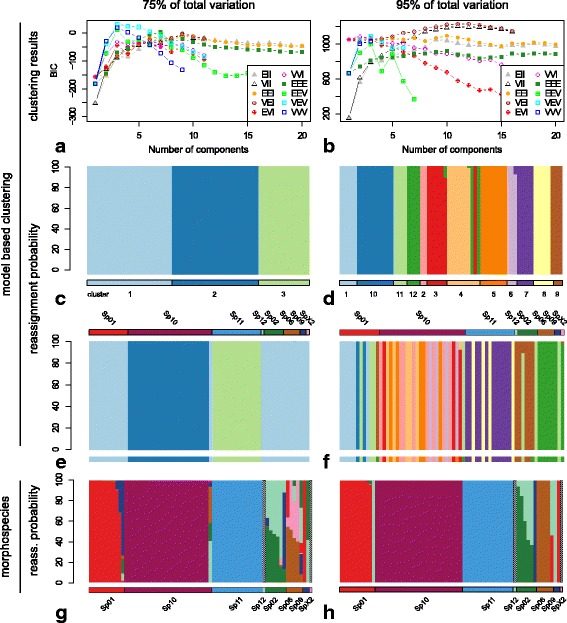
Species estimates of hierarchical clustering and confidence evaluation of *a priori* defined morphospecies and morphoclusters by LDA. Columns show results for 75 % and 95 % of total variation in the morphometric data. **a**, **b**: Choice of the best fitting cluster model by BIC. Reassignment probabilities to the clusters from hierarchical clustering with individuals ordered by (**c**, **d**) clusters and (**e**, **f**) by *a priori* defined morphospecies, and (**g**, **h**) reassignment probabilities to *a priori* defined morphospecies. Bars below plots C-H indicate prior group assignment for LDA, bars above plots E and F indicate affiliation to a priori defined morphospecies. Individuals in plots E-H are ordered identically

Hierarchical model-based cluster analysis [37] can identify unique morphological clusters of individuals without requiring *a priori* species assignments (e.g., [8]). The results of this analysis were extremely sensitive to the model choice (Fig. 3). Different mixture models favoured strikingly different numbers of clusters (e.g., 9, 7, 5, and 3 clusters were found for eigenshape axes 1–4 under different models) (BIC, Fig. 3a). The best model obtained for eigenshape axes 1–4 (the ellipsoidal, equal shape model; VEV) resulted in 3 clusters, but only morphospecies *sp11* and *sp10* (with the exception of one individual) were recovered as independent unique clusters. The best-fit model obtained for eigenshape axes 1–14 (the diagonal, varying volume, equal shape model; VEI) resulted in 12 clusters (Fig. 3b), with all morphospecies recovered in more than one group, with the exception of the singletons and *sp6*; the latter was recovered together with individuals of *sp9* and *spX2*.

Linear discriminant analysis (LDA) with respect to the *a priori* defined morphospecies recovered one of the eight species (*sp11*, 100 % of individuals) based on eigenshape axes 1–4 (Fig. 3, Additional file 1: Table S11). Two of the eight morphospecies were recovered with the LDA based on eigenshape axes 1–14 (*sp10, sp11,* 100 % of individuals; the remaining morphospecies were recovered for 50–92 % of individuals). LDA with respect to groups identified by the model-based cluster analysis recovered all three clusters correctly based on eigenshape axes 1–4 (Fig. 3, Additional file 1: Table S12). Finally, LDA on clusters from the second analysis based on eigenshape axes 1–14 recovered all but two of the groups for 100 % of individuals.

### Bayesian species delimitation

**Fig. 4:**
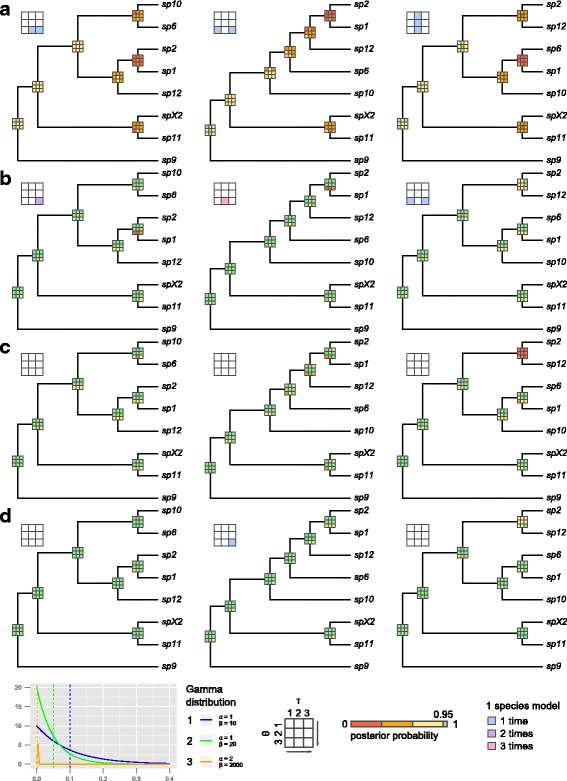
Mean posterior probabilities of Bayesian species delimitations from 10 repeated runs with commonly used priors. Means inferred under 9 different *θ* and *τ* prior combinations are color-coded in 3x3 boxes on each putative speciation split of the guide trees. The arrows in the legend point to the direction of more conservative prior choices. Columns from left to right: 3 alternative guide tree topologies from *BEAST, ML and MrBayes analyses, and a modified *BEAST topology based on morphological similarity of the species; rows: analyses using (**a**) no data (prior only), (**b**) molecular data, (**c**) morphometric trait data, and (**d**) both data sources. The colours of the large 3x3 inset boxes indicate the number of repeat-analyses that were stuck in the one species model. Gamma distribution densities of *θ* and τ priors 1–3 are depicted in the bottom left corner. Dashed lines indicate the respective distribution means

The total-evidence approach to Bayesian species delimitation [14, 34] provided strong support for the *a priori* defined morphospecies, however, for independent data types (molecular versus morphometric), the results were sensitive to the priors on the root age (*τ*_*0*_) and population size (*θ*) parameters (Fig. 4, Additional file 3: Table S13). Broadly, posterior probabilities (i.e., support for species delimitations) increased in the integrative analyses that combined molecular and morphological trait data (Fig. 4). While results were sensitive to both the choice of *τ*_*0*_ and *θ,* the choice of *θ* seemed to be more influential. The most consistent pattern that emerged is that low values of *θ* sometimes lead to low support for species delimitations. Species remained relatively well supported with high prior values of *τ*_*0*_. When the model was run under the prior (e.g., without data), with exception of the deepest divergences (*sp09*), the model did not result in any support (*P* > 0.95) for the *a priori* species assignments. This indicates that although the results were sensitive to the priors, the data contained informative signal.

Based on morphometric data alone, the divergence between *sp01* + *sp02* and *sp12* in tree A was strongly supported (*P* > 0.95), however, the combination with low *θ* values reduced support at these nodes (Fig. 4). The analysis based on molecular data alone provided overall support for the *a priori* morphospecies assignments. Exceptions occur for all nodes given low values of *θ* with all data sets. For example, *sp01* and *sp02* were strongly supported in analyses with higher values of *θ* (*P* > 0.95), while there was low support for this divergence in analyses with the lowest value of *θ* (*P* < 0.32). The delimitation between *sp02* and *sp12* (tree C) was the only split that consistently received low support under all *θ* prior values and with all data sets.

As expected, the results were also sensitive to the guide tree choice. For example, when *sp02* and *sp12* were specified as belonging to separate groups of species, they were always strongly supported with high posterior probabilities (tree A, B). However, when the guide topology was modified to accommodate the observed high morphological similarity between *sp02* and *sp12* (guide tree C), they were almost never recovered as independent species (Fig. 4). Interestingly, none of the *a priori* defined species gained high support for all prior combinations across all guide trees, even using the integrative total evidence approach (Fig. 4d).

The unguided analyses (molecular data only) that applied nearest neighbor interchanges (NNI) to the initial guide tree topologies largely confirmed the results of the guided (iBPP) analyses. While the initial guide tree and the choice of the *τ*_*0*_ prior did not alter the results, the choice of the *θ* prior had strong influence on the posterior probabilities of the speciation splits. All *a priori* defined morphospecies were well supported under *θ*_*1*_ and *θ*_*2*_ (Table 1) however, under the narrow and small *θ*_*3*_ prior, in particular *sp1* and *sp2*, but also *sp1, sp10, sp12, sp2,* and *sp6*, were lumped into one species (Additional file 4: Table S14 and Additional file 5: Table S15).

In the final set of analyses, in which *sp10* was specified as two separate entities, corresponding to two distinct genotypes (Additional file 1: Figure S7), this split was not supported based on the analysis of the morphometric data alone, as expected. However, this split received strong support based on the analysis of both the molecular only and combined datasets (Additional file 1: Figure S7).

## Discussion

### Congruence between single DNA markers and morphometric evidence

Using a wide range of morphometric and phylogenetic tools, we tested for congruence between morphological, molecular, and integrative approaches (i.e., iterative *sensu* [19]) to species delimitation in the chafer beetle genus *Pleophylla*. Morphometric analysis (eigenshape analysis) of the left paramere of the male genitalia, as well as subsequent CVA and LDA provided quantitative support for the majority species assignments based on morphology. In contrast, model based hierarchical clustering showed much less congruence with the morphospecies (Fig. 3e, f), indicating that this approach may not be suitable for delimitation at the level of species.

Molecular-based species delimitation resulted in a wide range of support for morphospecies, based on the analysis of standard markers used among beetles (e.g., [82–85]), from zero (28S and *rrnL*) to moderate or high (*cox1* and ITS1). The ribosomal markers were insufficiently informative to support any of the putative morphospecies (Additional file 1: Figure S3-S5; Table S8), due to the remarkably low interspecific molecular variation observed across the entire genus. This is less surprising for the slowly evolving 28S rRNA marker, but *rrnL* has previously provided reasonable resolution at the species level among scarabs (e.g., [82]). The mitochondrial gene *cox1* and the nuclear region ITS1 were more informative, while the latter provided the best resolution. There was overall congruence between the morphospecies and the ITS1 MOTUs (GMYC, ABGD, PTP), despite the fact that ITS1 had fewer haplotypes than *cox1* (23 versus 53) and a lower relative substitution rate (Additional file 1: Table S5). A wide range of tree building methods, parameters and tree linearization approaches did not improve the results of the GMYC model using *cox1*. In particular, there were three putative morphospecies that were difficult to distinguish on the basis of molecular data alone (*sp01* vs *02; sp6* vs *sp10; sp02* vs *sp12*). At one extreme, individuals belonging to a single morphospecies (*sp10*) were assigned to two MOTUs on the basis of two distinct ITS1 genotypes. The genotypes had a total of 31 segregating sites, including one 2-base-deletion, two 4-base-deletions, and one 2-base-insertion, indicating that a single mutation is unlikely to the cause of the molecular variation, although this pattern was not recovered by any other marker. At the other extreme, individuals belonging to two distinct morphospecies were assigned to a single MOTU and shared identical *cox1* and ITS1 haplotypes (*sp01*, *sp02*), which may be attributed to introgressive hybridisation or incomplete lineage sorting.

Distinguishing between secondary gene flow and incomplete lineage sorting is difficult because both processes produce similar phylogenetic patterns [66]. JML analyses [67] indicated that incomplete lineage sorting may be sufficient to explain the observed level genetic variation across species with independent data partitions and species – with the exception of the fast evolving *cox1* third codon (Additional file 1: Table S5; *sp09* and *sp11*), the monophyly of these species was otherwise well supported*.* The basic substitution model implemented in JML may not be sufficient to account for hidden substitutions at this site and may underestimate the genetic distance for this partition (Additional file 1: Table S5, S7). Overall, the JML results provide support for an incomplete lineage sorting scenario, however, this test cannot be treated as definitive against secondary gene flow. The method implemented in JML can only be used to detect hybridization events for sequences that have a coalescence time younger than the speciation event [66], and this approach can result in false negatives [86].

### Bayesian species delimitation using an integrative taxonomy framework

In concordance with our results from the Bayesian species delimitation, Solís-Lemus et al. [14] have shown that the integration of morphological evidence together with molecular data may greatly enhance the discriminative power of species delimitation models. However, it has also been shown that errors and uncertainties in upstream analyses (e.g., guide tree inference, individual-species assignment) and prior parameter choice may impact the accuracy of results [81, 87, 88]. Here, we assessed the impact of a wide range of parameter combinations, including prior parameter and guide tree choice.

Leaché and Fujita [81] previously demonstrated the significant impact of using randomly generated guide tree topologies. Rannala [89] questioned the practicality of exhaustive guide tree manipulation, with respect to the increased computation time associated with popular phylogenetic inference methods. In addition, a random set of guide trees will include some unreasonable or unlikely topologies, which can lead to inaccurate delimitations (e.g., over-splitting; [88]). Here, we limited our guide tree choice to three options, justified on the basis of evidence of independent molecular and morphometric evidence, in order to further evaluate incongruences between both data sources (Additional file 1: Figure S2). The use of alternative guide trees had a large impact on the results. For example, the use of guide tree C (based on morphological similarity) allowed us to identify support for a putative species pair (*sp02*/*sp12*), which was otherwise not identified using alternative molecular based approaches, including the unguided (NNI) approach in BPP (Additional file 4: Table S14 and Additional file 5: Table S15). In an additional set of experiments, we used a fourth guide tree topology based on the support for a putative case of cryptic diversity obtained using alternative single-marker delimitation approaches (*sp10*, Additional file 2: Table S9; Additional file 1: Figure S2, S7). This experiment, however, cannot provide definitive support for these species entities, because the units were inferred on the basis of non-independent evidence. Manual inspection of the alignments for *sp10* revealed 2 ITS1 genotypes with 43 segregating sites represented by *sp10a* and *sp10b*. This is a very strong signal compared to a total of 44 segregating sites in both mitochondrial markers, which did not exhibit any diverging signal between *sp10a* and *sp10b*. Only a single site was polymorphic for *sp10b* in 2 of the 4 *sp10b* specimens. However, these analyses serve to demonstrate that the results obtained using this model can be extremely sensitive to the signal present in single molecular markers, even in presence of data that provide strong evidence for morphological similarity (Additional file 1: Figure S7).

The use of alternative prior combinations for the population size (*θ*) and root age (*τ*) priors each had a large impact on the results. These analyses indicate that these parameters must either be chosen using extreme caution (using independent empirical evidence) or multiple analyses should be performed to assess the robustness of species delimitations to these parameters, such as the analyses performed here. We found that phylogenetically younger species (*sp01*, *sp02*, *sp06*, *sp10, sp12*) and analyses that employed less data (e.g., single versus combined traits) were typically more sensitive to the results. It has also been demonstrated that strong variation in mutation rate and population size among populations or species can also decrease the accuracy of alternative coalescent-based delimitation models [90].

The inclusion of more individuals (and/or data) can lead to more accurate and precise parameter estimates [91], but increased taxon sampling is sometimes not possible due to the natural rarity of some species [73]. The development of better approaches to account for this uncertainty may be important, because in reality many biodiversity studies will be subject to limited taxon sampling. Further research using empirical and simulated data are required to fully assess the impact of guide tree, prior parameter choice, model violation and taxon sampling. Here, we demonstrate that the inclusion of morphological data can lead to more robust estimates of species delimitations. The results obtained using the combined dataset are less sensitive to prior parameter choice, than the analysis based on molecular or morphological dataset alone (Fig. 4; Additional file 3: Table S13, Additional file 4: Table S14 and Additional file 5: Table S15). Overall, nearly all morphospecies received strong support based on the analysis of the combined dataset (Fig. 4d). All sequence-based inference methods, including tree inference using concatenated data or coalescent-based approaches such as *BEAST and BPP, may be impacted by putative incomplete lineage sorting or introgression. An integrative approach to taxonomy enables all available evidence to be utilized and may be particularly useful for delimitating very young species, which will always be difficult to distinguish on the basis of molecular or morphological data alone.

### Conclusions

The earliest stages of speciation will be the point at which it will be the hardest to establish a boundary between population and species level divergence. However, such cases (and their solution) are the “holy grail” of taxonomy and provide an exemplar for investigating the intermediate stages of the “Darwinian continuum” from varieties to species [92] and inevitably create problems for the definition of species. Integrative or multiple strategies may be necessary in such cases where conflicts are most likely to exist [6, 19]. Together with previous studies [7, 14] we have confirmed that morphology can be a highly informative trait within an integrative approach, such as iBPP, to species delimitation.

Complex cases of species delimitation, such as those among *Pleophylla* species, demonstrate the sensitivity of delimitation approaches to prior parameter choices and are thus useful for investigating the performance of new methodologies. We have highlighted the importance of examining the effect of prior choice on species delimitation results in BPP and iBPP, especially if highly informative prior distributions (α >1) are used. Previously, specifying a high *θ* and a low *τ*_*0*_ value was intended to constitute a conservative prior combination that should not lead to over-splitting [81]. However, we found that this combination actually led to higher support for more splits, which was attributable to the strong influence of the *θ* parameter. For a conservative estimate of species delimitations, we recommend using a low value of *θ* to avoid species over-splitting.

**Fig. 5:**
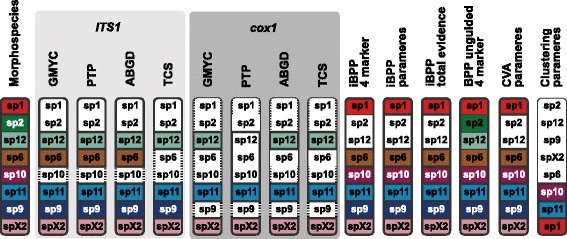
Overview of the results from the different species delimitation methods and data. Inferred entities that were fully congruent with the *a priori* morphospecies assignments are indicated by the bold circumscribed coloured squares, incongruent units remain white; sub-splitting within morphospecies is indicated by horizontal dashes. Additional sub-splitting within morphospecies that share overlapping MOTUs are circumscribed by a narrow line. Uncertain delimitations are indicated by thin lines between *a priori* morphospecies

The incongruence between trait- or gene-based species delimitations (Fig. 5, Additional file 1: Figure S8) may have multiple independent causes. First, sampling issues and the ability to capture statistically significant entities may be problematic, particularly for trait-based inference [7] (see also above). For example, trait-based clustering algorithms quickly loose power when including too many or too poorly sampled species, or when variation is distributed over too many dimensions, resulting in more noise [14, 93]. These problems may also pose a challenge for combined approaches to species delimitation, however their impacts have not been fully explored. Second, the incongruence among independent methods, employed for the analysis of different data types (molecules versus discrete or continuous morphological traits), may be attributed to the use of competing species concepts [94, 95]. Model based clustering applied on morphological traits is simply based on the morphological species concept; tree-based species inference methods (e.g., GMYC, PTP) are based on the phylogenetic species concept in [88, 96], which rely on the assumption of reciprocal monophyly across gene trees. The assumption of monophyly among independent markers may be problematic because this assumption is known to be violated for closely related species. de Queiroz redefined the criteria inherent to most species concepts [21, 97, 98] that species represent independent metapopulation lineages through time. Instead, in the generalized lineage concept (GLC) the criteria used to demarcate species (e.g., morphological differences, monophyly or reproductive isolation) are treated as attributes that accumulate during the process of lineage diversification [98]. This concept has been broadly adopted by coalescent-based approaches to species delimitation [6, 7, 10, 20, 34, 99–103], which model the lineage diversification process using multiple markers to delimit species (e.g., [104]). Several studies have delimited species successfully using these approaches [5, 81, 94, 95, 105, 106].

BPP (and iBPP) treat species as hypotheses in a probabilistic framework, using objective tests to delineate independent evolutionary lineages (i.e., species), therefore satisfying numerous species concepts [95]. Caution should always be taken when interpreting the results of a single dataset [6, 7], however, an integrative model-based approach to detecting species is likely to have more utility and could result in more robust species delimitations, especially when divergence varies across different phenotypic, genetic or ecological parameters [7].

Finally, based on the outcome of the integrative BPP analysis (Figs. 4 and 5), which was broadly congruent with the single trait evidence, we conclude that in our *Pleophylla* data set *sp1*, *sp6*, *sp9*, *sp10, sp11*, and *spX2* are valid species, while *sp2* and *sp12* very likely belong to the same taxon. The results of alternative molecular delimitation methods provided support for potential cryptic species (*sp10,* Additional file 1: Figure S8). However, this signal comes from one of the four markers only (which we demonstrated can overwhelm the signal of other data in the BPP/iBPP analyses, Additional file 1: Figure S7) and this result is not corroborated by morphological or geographical evidence (the two MOTUs occur in the same location). Therefore, at this stage we do not consider these as two separate species. (These conclusions will be further developed by formal taxonomic treatment, type material and taxonomic revision that will be presented in a separate upcoming study; Eberle J, Beckett M, Özguel-Siemund A, Frings J, Fabrizi S, Ahrens D. Afromontane forests hide nineteen new species of ancient *Pleophylla *chafers (Coleoptera: Scarabaeidae): phylogeny and taxonomic revision, in preparation). Additional information about the structure of a population or species complex, based on much broader individual, geographical and DNA sequence sampling would very likely have improved our case study. However, natural rarity (linked with the time constraints of most biodiversity studies) will always have an impact on the number of available samples and may strongly bias the results [107].

Simulations have suggested that the number of loci required for robust Bayesian species delimitation may be large [10]. Here, we demonstrate that the signal from a single marker can influence the outcome of a fully integrative analysis, even given the inclusion of morphology. These results further underlay the necessity for upgrading the globally successful Barcoding initiatives to include a broader range of universal markers (e.g., [108]). Despite numerous disadvantages [109, 110], this approach would help to overcome some of the major challenges to accurate species delimitation [111]. Future directions in integrative taxonomy will need to further address these issues, including integrative study design and the interpretation of frequently incongruent results. In addition the development of new tools for integrating disparate types of specimen-based data in taxonomic studies offer an exciting opportunity to free taxonomy from subjectivity.

### Availability of supporting data

Voucher specimens have been deposited in the Zoological Research Museum A. Koenig (Bonn). All molecular sequences generated for this study were deposited in GenBank (Additional file 1: Table S1). Sequence alignments, program input files and phylogenetic trees were deposited on Zenodo (doi:10.1186/s12862-016-0659-3) [112]. The perl script used for running (i)BPP with multiple prior combinations, along with all input files, is available at https://github.com/eberlejonas/BPPmulti.git.

## Additional files

Additional file 1: Supplementary text, figures S1-S8, tables S1-S8, S10-S12. (PDF 1909 kb) Additional file 2: 3d morphospace. (GIF 6172 kb) Additional file 3: Supplementary table S9. (XLSX 64 kb) Additional file 4: Supplementary table S13. (XLS 185 kb) Additional file 5: Supplementary table S14. (XLS 40 kb) Additional file 6: Supplementary table S15. (XLS 38 kb)
