# Supplementary material for: Bayesian species delimitation in Pleophylla chafers (Coleoptera) – the importance of prior choice and morphology
Source: BMC Evol Biol. 2016 May 5;16:94. doi: 10.1186/s12862-016-0659-3 (PMC4858874; doi:10.1186/s12862-016-0659-3)
Supplement: Additional file 1: — Supplementary text, figures S1-S8, tables S1-S8, S10-S12. (PDF 1909 kb) [file 12862_2016_659_MOESM1_ESM.pdf]

Supplementary materials and methods

Taxon sampling and DNA sequencing

110 individuals of the genus *Pleophylla* were collected from eight localities in South Africa (Supplementary Table S1-S2). Samples were preserved in ethanol, and following DNA extraction, individuals were dry mounted and preserved for morphological study. DNA was extracted non-destructively from leg or thorax tissue using the Promega Wizard SV96 Plate extraction kit, as per the manufacturer's protocol. An 826 bp fragment of the 3' end of the mitochondrial gene cytochrome oxidase subunit 1 (*cox1*) was amplified using primers C1-J-2183 (Jerry) and TL2-N-3014 (Pat) (Simon et al., 1994). A 469 – 471 bp fragment of the mitochondrial 16S rRNA gene (*rrnL*) was amplified using the primer LR-N-13398 (16Sar) paired with either N1-J-12585 (ND1A) or LR-J-12961 (16Sb2) [1]. A 645 bp fragment of the nuclear ribosomal rRNA 28S gene was amplified using the primers 28SFF and 28SDD [2]. The nuclear internal transcribed spacer 1 (ITS1) (636 – 723 bp) was amplified using the primers 5' GTAGGTGAACCTGCAGAAGG and 5' GCGTTCGAARTGCGATGATCAA [3]. These primers are sometimes referred to as ITS1R and ITS1F, respectively (e.g., [4, 5]), but note that ITS1R binds to the 5' 18S region, while ITS1F binds to the 3' 5.8S region of this product.

Amplified products were sequenced in both directions using ABI BigDye technology and an AB1 PRISM 3730 DNA Analyzer (Applied Biosystems) at the Natural History Museum (London). Contiguous sequences were assembled from both strands and edited using Sequencher 4 (Gene Codes Corporation, Ann Harbor, MI, USA). All sequences were deposited in GenBank. Accession numbers, identifications and specimen

vouchers are provided in Supplementary Table S1.

Details of the morphometric analysis

The partial outline of the male's left paramere was digitized, extracted from specimens using tpsDig 2.1 [6], and subsequently sampled as a set of 150 semi-landmarks. Digitised curves of the male's left paramere (Figure S1) were analysed using Morpho-tools [7]. Sets of outline coordinate points were converted from the Cartesian (x,y) form to the φ form of Zahn and Roskies' [8] shape function, removing size information. The resultant shape functions were expressed as the raw net angular deviation between outline coordinates. The shapes were mean centered and no standardization was applied. Standard Eigenshape analysis [9, 10] was performed in Eigenshape 2.6, as implemented in Morpho-tools, using the covariance for calculation of the similarity matrix and φ.

Sequence alignment and model selection

Cox1, rrnL and 28S sequences were aligned unambiguously using ClustalW 2 [11], with default gap opening and extension penalties (15 and 6.66 respectively). ITS1 was aligned using ClustalW, with further refinement using the refine option in MUSCLE [12]. The ITS1 alignment was further edited, and regions that could not be unambiguously aligned were removed. For all remaining sequences, the optimal partition schemes and substitution models were simultaneously identified using the Bayesian Information Criterion implemented in PartitionFinder [13], with branch lengths linked across

partitions. It has been demonstrated that it is difficult to estimate the gamma distribution and invariant sites parameters simultaneously [14-16]. To avoid over-parameterization, these parameters were never combined in the same model.

Details of phylogenetic analyses and divergence time estimation

Unpartitioned maximum likelihood analysis was performed using the subtree pruning and regrafting (SPR) algorithm implemented in PhyML 3.0 [17]. All model parameters were estimated, and where applicable, we implemented 5 gamma rate categories. The Bayesian-like transformation of the approximate likelihood ratio test (aBayes) was used to assess branch support [18].

Partitioned maximum likelihood analysis was performed using RAxML 7.3 [19, 20], implementing the rapid bootstrapping algorithm and a subsequent search for the best scoring ML tree. The number of bootstrap replicates for each dataset was determined using the bootstopping criteria [21], with a maximum of 1,000 replicates.

Bayesian phylogenetic analysis was performed using the MPI version of MrBayes 3.1.2 [22-24]. Branch lengths were linked across partitions but partitions were allowed to evolve under different rates. All other substitution model parameters were unlinked across partitions. It has been demonstrated that the default prior on branch lengths implemented in MrBayes can lead to spuriously large estimates of internal branch lengths [25], and consequently the 95% posterior credibility intervals can exclude the maximum likelihood estimate for tree length [26]. The default branch length prior in MrBayes is an exponential distribution with rate parameter $\lambda = 10$, where the mean = 0.1

substitutions/site. Analysis of our dataset using the default branch length prior in MrBayes produced estimates of total tree length (sum of branch lengths) that were an order of magnitude greater than the maximum likelihood estimate obtained in RAxML and PhyML. Because the GMYC approach to species delineation is sensitive to estimates of branch lengths, we ran four sets of analyses using an exponential prior on the branch lengths with mean = 0.1 (default), 0.05, 0.01 or 0.005. Each analysis included two parallel runs of 10 million generations, using one cold and three incrementally heated Markov chains ($\lambda = 0.1$), sampling every 1,000 steps and discarding the first 3,000 trees as burn-in. Convergence and mixing were assessed using standard diagnostics (standard deviation of split frequencies, effective sample size, and visual inspection of trace plots). All MCMC output was examined visually using Tracer 1.5 [27].

The species tree and individual gene trees (*cox1* and ITS1) were co-estimated using the multispecies coalescent model implemented in *BEAST 1.75 [28, 29]. This analysis requires species (or operational taxonomic units) to be defined *a priori*. We used the putative morphospecies to define taxonomic units and omitted all 14 individuals for which there was ambiguity (Supplementary Table S1). Outgroups were not included in this analysis. We implemented the partition scheme and partition-specific models selected for both markers using PartitionFinder for analysis in MrBayes (*cox1* [P1 vs. P2 vs. P3] and ITS1). Substitution model parameters were estimated separately for each partition. Base frequencies were calculated from the data. The transition-transversion ratio parameter of the HKY substitution model was specified using a lognormal prior with mean and standard deviation equal to 1 and 1.25, respectively, *LN*(1, 1.25). The exchangeability rate parameters of the GTR model were specified using a gamma prior,

with shape and scale parameters equal to 1, *G*(1,1). The invariant sites parameter was specified using a uniform prior, *U*(0, 1). Rate heterogeneity across sites was modelled using the discrete gamma model, with four independent rate categories and the shape parameter was specified using an exponential prior, with mean equal *E*(0.5).

The uncorrelated lognormal relaxed clock model was implemented to estimate divergence times [30]. The mean substitution rate of *cox1* was fixed, and a diffuse exponential prior (*E*(1/3)) was used to specify the parameter that describes variation in the substitution rate (ucld.stdev). Clock model parameters were unlinked across genes, and the rate of ITS1 was estimated relative to that of *cox1*. We applied a range of mean branch rates, in five independent sets of analyses (2, 2.5, 3, 3.5 or 4% My^{-1}). The parameter used to describe rate variation across branches in the relaxed clock model – the standard deviation of the lognormal distribution of rates – was specified using an exponential prior, *E*(0.3). The mean of *cox1* was fixed as described above, and the mean rate of ITS1 was specified using an exponential prior, *E*(1).

We assumed a Yule speciation process, with a constant speciation rate and population size through time. The population size and birth rate parameters were specified using the non-informative improper prior, 1/*x*. Two independent MCMC runs were performed for each analysis, each consisting of 500 million iterations, sampling every 5,000th generation and discarding the first 50 million steps, resulting in 90,000 samples post burn-in. As above, convergence was assessed using Tracer 1.5. Mean node ages and credibility intervals were calculated using TreeAnnotator 1.7.5.

JML simulations

JML [40, 41] calculates the expected minimum pairwise distances under a coalescent model that assumes no migration (or hybridization), for a given set of individuals, by simulating gene trees and sequence datasets – this requires estimates of topology, population size and temporal branch lengths. To account for uncertainty in the estimation of these parameters, simulations were performed using 10,000 trees from the posterior distribution of species trees output by each of the *BEAST analyses, which include estimates of population size and branch lengths. Analysis in JML was performed individually for each partition specified in the *BEAST analysis (*cox1* [P1 vs. P2 vs. P3] and ITS1). The substitution model and rate parameters were specified using the parameters estimated by *BEAST. The relative rates for each *cox1* partition were calculated by multiplying the relative rate of each codon by the mean branching rate of *cox1*. Appropriate heredity scalars were selected for *cox1* (= 0.5) and ITS1 (= 2).

Supplementary results

Sequence data, alignment and model selection

We obtained 438 new sequences for 110 individuals (Supplementary Table S1). There is remarkably low molecular variation among the members of the genus. Excluding outgroups, the final alignments for each individual marker contained 173, 18, 3 and 194 parsimony informative characters for *cox1* (826 bp), *rrnL* (469 – 471 bp), 28S (645 bp) and ITS1 (726 – 812 bp), respectively. Previously published *cox1*, *rrnL* and 28S sequences for the outgroup species (*Omalopia nigromarginata* and *O. ruricola*) were

included in our analysis [42]. We also obtained ITS1 sequences for *O. nigromarginata* and *O. ruricola*, however it was not possible to align unambiguously these sequences with the ingroup taxa, so these sequences were excluded from further analysis. The final concatenated alignment contained 2,795 characters, including 3.57% missing data and gaps. The partition schemes and optimal substitution models selected using PartitionFinder for each dataset and for use in different programs are presented in Supplementary Table S3.

Phylogenetic analysis and the monophyly of morphospecies

Morphospecies *sp09* was the only species that was ubiquitously recovered as monophyletic in the independent analyses of all four markers. In the analysis of *rrnL* specimens *Pleo*834751 (*sp10*) and *Pleo*834776 (*sp11*) were recovered within the *sp11* and *sp06* clades respectively, probably due to misleading phylogenetic signal or laboratory errors. Otherwise there was consistently good support for the monophyly of *sp11*. Morphospecies *sp10* and *sp06* were not recovered as monophyletic by *cox1*, but these groups received strong support in both the ITS1 gene tree and the combined analyses. The clade that contains morphospecies *spX2* + five female specimens was also strongly supported.

The interspecific relationships were also strongly supported in the combined analysis. Morphospecies *sp09* was always recovered as the most ancestral branching member of the genus. The next most ancestral branching group was morphospecies *sp12* + *sp11*, however the position of morphospecies *sp12* fluctuates in the *rrnL*, *cox1* and

combined mitochondrial trees (Figs. S3-S5). The interspecific relationships among morphospecies *sp09*, *sp11*, *sp10*, *sp06*, *spX2* and the morphospecies *sp01*/ *sp02* group were fully resolved and strongly supported in the combined analysis using different tree inference approaches (Figs. S3-S5).

The Bayesian species tree estimated using *BEAST for the combined *cox1* and ITS1 dataset resulted in strong support for the same interspecific relationships estimated using the *cox1* data. Individual gene trees (for *cox1* and ITS1) shared the same topology as the gene trees estimated using PhyML, RAxML and MrBayes, but in contrast, the species tree favoured a sister group relationship between *sp06* and *sp10*. The estimated divergence times for each species pair and associated uncertainty obtained under variable substation rates are presented in Supplementary table 6.

The impact of the branch length prior in MrBayes

Changing the branch length prior implemented in MrBayes had no impact on the inferred topology but had a large impact on tree length (the sum of branch lengths) (Supplementary Table S4). Note that in some cases the Bayesian 95% posterior intervals do not contain the maximum likelihood estimate. A large difference was observed between the estimates of tree length obtained using the Bayesian analysis, under the default branch length prior in MrBayes, and the maximum likelihood estimates obtained using RAxML and PhyML (Supplementary Table S4). Changing the branch length prior to favour shorter branch lengths reduced this discrepancy, and for analyses of *cox1* actually produced shorter lengths than the maximum likelihood analyses. Partitioned

maximum likelihood analysis produced longer tree lengths. In particular, when *cox1* is included in the analysis, partitioning by codon had a large impact on the branch lengths.

JML – results obtained for independent partitions

The observed pairwise distances between individuals of all morphospecies were not lower than expected at the 5% level ($P > 0.05$), given the null model (the coalescent with no migration or hybridization). However, the results obtained using JML varied among partitions (Supplementary Table S7). The minimum distances and the probabilities of observing these distances were similar for the first and second (*cox1*) codon partitions ($P > 0.1$ for all pairwise comparisons). In contrast, the minimum distances were much greater for the third (*cox1*) codon partition, and the probabilities of obtaining the observed distances under the null model were smaller. The probability of obtaining the observed pairwise distances between morphospecies *sp09* or *sp11* and all other species was lower than expected at the 10% level ($P < 0.1$; $P > 0.1$ for all other pairwise comparisons). Note that the third codon position evolves at one and two orders of magnitude faster than the first and second positions respectively (Supplementary Table S5). For the ITS1 data, the probabilities of obtaining the observed sequence distances under the null model were not lower than expected ($P > 0.2$ for all pairwise comparisons).

References

1. Simon C, Frati F, Beckenbach A, Crespi B, Liu H, Flook P: **Evolution, Weighting, and Phylogenetic Utility of Mitochondrial Gene-Sequences and a Compilation of Conserved Polymerase Chain-Reaction Primers**. *Ann Entomol Soc Am* 1994, **87**(6):651-701.
2. Monaghan MT, Inward DJG, Hunt T, Vogler AP: **A molecular phylogenetic analysis of the Scarabaeinae (dung beetles)**. *Mol Phylogenet Evol* 2007, **45**(2):674-692.
3. Vogler AP, Desalle R: **Evolution and Phylogenetic Information-Content of the Its-1 Region in the Tiger Beetle Cicindela Dorsalis**. *Mol Biol Evol* 1994, **11**(3):393-405.
4. Kerdelhue C, Roux-Morabito G, Forichon J, Chambon JM, Robert E, Lieutier F: **Population genetic structure of Tomicus piniperda L. (Curculionidae : Scolytinae) on different pine species and validation of T-destruens (Woll.)**. *Mol Ecol* 2002, **11**(3):483-494.
5. Santos H, Rousselet J, Magnoux E, Paiva MR, Branco M, Kerdelhue C: **Genetic isolation through time: allochronic differentiation of a phenologically atypical population of the pine processionary moth**. *P Roy Soc B-Biol Sci* 2007, **274**(1612):935-941.
6. Rohlf FJ: **TPSDig 2.1.** http://www.life.bio.sunysb.edu/morph/ 2006.
7. Krieger JD: **Measure LMs 4.0. Morpho-tools** http://www.morpho-tools.net 2006.
8. Zahn CT, Roskies RZ: **Fourier Descriptors for Plane Closed Curves**. *Ieee T Comput* 1972, **C 21**(3):269-&.
9. MacLeod N: **Generalizing and extending the eigenshape method of shape space visualization and analysis**. *Paleobiology* 1999, **25**(1):107-138.
10. Macleod N, Rose KD: **Inferring Locomotor Behavior in Paleogene Mammals Via Eigenshape Analysis**. *Am J Sci* 1993, **293a**:300-355.
11. Larkin MA, Blackshields G, Brown NP, Chenna R, McGettigan PA, McWilliam H, Valentin F, Wallace IM, Wilm A, Lopez R *et al*: **Clustal W and clustal X version 2.0**. *Bioinformatics* 2007, **23**(21):2947-2948.
12. Edgar RC: **MUSCLE: multiple sequence alignment with high accuracy and high throughput**. *Nucleic Acids Res* 2004, **32**(5):1792-1797.
13. Lanfear R, Calcott B, Ho SYW, Guindon S: **PartitionFinder: Combined Selection of Partitioning Schemes and Substitution Models for Phylogenetic Analyses**. *Mol Biol Evol* 2012, **29**(6):1695-1701.
14. Mayrose I, Friedman N, Pupko T: **A Gamma mixture model better accounts for among site rate heterogeneity**. *Bioinformatics* 2005, **21**:151-158.
15. Sullivan J, Swofford DL, Naylor GJP: **The effect of taxon sampling on estimating rate heterogeneity parameters of maximum-likelihood models**. *Mol Biol Evol* 1999, **16**(10):1347-1356.
16. Yang ZH: **Maximum-Likelihood-Estimation of Phylogeny from DNA-Sequences When Substitution Rates Differ over Sites**. *Mol Biol Evol* 1993, **10**(6):1396-1401.
17. Guindon S, Dufayard JF, Lefort V, Anisimova M, Hordijk W, Gascuel O: **New Algorithms and Methods to Estimate Maximum-Likelihood Phylogenies: Assessing the Performance of PhyML 3.0**. *Syst Biol* 2010, **59**(3):307-321.
18. Anisimova M, Gil M, Dufayard JF, Dessimoz C, Gascuel O: **Survey of Branch Support Methods Demonstrates Accuracy, Power, and Robustness of Fast Likelihood-based Approximation Schemes**. *Syst Biol* 2011, **60**(5):685-699.
19. Stamatakis A: **RAxML-VI-HPC: Maximum likelihood-based phylogenetic analyses with thousands of taxa and mixed models**. *Bioinformatics* 2006, **22**(21):2688-2690.
20. Stamatakis A, Hoover P, Rougemont J: **A Rapid Bootstrap Algorithm for the RAxML Web Servers**. *Syst Biol* 2008, **57**(5):758-771.
21. Pattengale ND, Alipour M, Bininda-Emonds ORP, Moret BME, Stamatakis A: **How Many Bootstrap Replicates Are Necessary?** *J Comput Biol* 2010, **17**(3):337-354.
22. Altekar G, Dwarkadas S, Huelsenbeck JP, Ronquist F: **Parallel metropolis coupled Markov chain Monte Carlo for Bayesian phylogenetic inference**. *Bioinformatics* 2004, **20**(3):407-415.
23. Huelsenbeck JP, Ronquist F: **MRBAYES: Bayesian inference of phylogenetic trees**. *Bioinformatics* 2001, **17**(8):754-755.

24. Ronquist F, Huelsenbeck JP: **MrBayes 3: Bayesian phylogenetic inference under mixed models**. *Bioinformatics* 2003, **19**(12):1572-1574.
25. Marshall DC: **Cryptic Failure of Partitioned Bayesian Phylogenetic Analyses: Lost in the Land of Long Trees**. *Syst Biol* 2010, **59**(1):108-117.
26. Rannala B, Zhu TQ, Yang ZH: **Tail Paradox, Partial Identifiability, and Influential Priors in Bayesian Branch Length Inference**. *Mol Biol Evol* 2012, **29**(1):325-335.
27. Drummond AJ, Rambaut A: **BEAST: Bayesian evolutionary analysis by sampling trees**. *Bmc Evol Biol* 2007, **7**.
28. Drummond AJ, Suchard MA, Xie D, Rambaut A: **Bayesian Phylogenetics with BEAUti and the BEAST 1.7**. *Mol Biol Evol* 2012, **29**(8):1969-1973.
29. Heled J, Drummond AJ: **Bayesian Inference of Species Trees from Multilocus Data**. *Mol Biol Evol* 2010, **27**(3):570-580.
30. Drummond AJ, Ho SYW, Phillips MJ, Rambaut A: **Relaxed phylogenetics and dating with confidence**. *Plos Biol* 2006, **4**(5):699-710.
31. Fontaneto D, Herniou EA, Boschetti C, Caprioli M, Melone G, Ricci C, Barraclough TG: **Independently evolving species in asexual bdelloid rotifers**. *Plos Biol* 2007, **5**(4):914-921.
32. Pons J, Barraclough TG, Gomez-Zurita J, Cardoso A, Duran DP, Hazell S, Kamoun S, Sumlin WD, Vogler AP: **Sequence-based species delimitation for the DNA taxonomy of undescribed insects**. *Syst Biol* 2006, **55**(4):595-609.
33. Britton T, Anderson CL, Jacquet D, Lundqvist S, Bremer K: **Estimating divergence times in large phylogenetic trees**. *Syst Biol* 2007, **56**(5):741-752.
34. Sanderson MJ: **r8s: inferring absolute rates of molecular evolution and divergence times in the absence of a molecular clock**. *Bioinformatics* 2003, **19**(2):301-302.
35. Rambaut A, Charleston M: **TreeEdit 1.0.** In.: http://tree.bio.ed.ac.uk/software/treeedit/ 2002.
36. Ezard THG, Fujisawa T, Barraclough TG: **SPLITS: Species' Limits by Threshold Statistics R package**. http://barralab.bio.ic.ac.uk 2009.
37. Powell JR: **Accounting for uncertainty in species delineation during the analysis of environmental DNA sequence data**. *Methods Ecol Evol* 2012, **3**(1):1-11.
38. Templeton AR, Crandall KA, Sing CF: **A Cladistic-Analysis of Phenotypic Associations with Haplotypes Inferred from Restriction Endonuclease Mapping and DNA-Sequence Data .3. Cladogram Estimation**. *Genetics* 1992, **132**(2):619-633.
39. Clement M, Posada D, Crandall KA: **TCS: a computer program to estimate gene genealogies**. *Mol Ecol* 2000, **9**(10):1657-1659.
40. Joly S: **JML: testing hybridization from species trees**. *Mol Ecol Resour* 2012, **12**(1):179-184.
41. Joly S, McLenachan PA, Lockhart PJ: **A Statistical Approach for Distinguishing Hybridization and Incomplete Lineage Sorting**. *Am Nat* 2009, **174**(2):E54-E70.
42. Ahrens D, Vogler AP: **Towards the phylogeny of chafers (Sericini): Analysis of alignment-variable sequences and the evolution of segment numbers in the antennal club**. *Mol Phylogenet Evol* 2008, **47**(2):783-798.

Table S1. Accession numbers for specimens included in the morphometric and phylogenetic analyses, along with voucher numbers and geographical origin. All specimens were included in the phylogenetic analyses. Specimens that were included in the morphometric analysis are indicated by an asterisk (*).

Morphospecies	Voucher	Locality	*cox1*	*rrnl*	ITS1	28S
Omaloplia nigromarginala	747065	-	EF487770	EF487791	EU084255	NA
Omaloplia ruricola	747063	-	EF487771	EF487790	EU084256	NA
Pleophylla sp 1	834691	L1	KC904098	KC964429	KC964210	KC964320
Pleophylla sp 1	834692*	L1	KC904099	KC964430	KC964211	KC964321
Pleophylla sp 1	834694*	L1	KC904101	KC964432	KC964213	KC964323
Pleophylla sp 1	834699*	L1	KC904106	KC964437	KC964218	KC964328
Pleophylla sp 1	834700	L1	KC904107	KC964438	KC964219	KC964329
Pleophylla sp 1	834701*	L1	KC904108	KC964439	KC964220	KC964330
Pleophylla sp 1	834703	L1	KC904110	KC964441	KC964222	KC964332
Pleophylla sp 1	834704	L1	KC904111	KC964442	KC964223	KC964333
Pleophylla sp 1	834706	L1	KC904113	KC964444	KC964225	KC964335
Pleophylla sp 1	834708*	L1	KC904115	KC964446	KC964227	KC964337
Pleophylla sp 1	834709*	L1	KC904116	KC964447	KC964228	KC964338
Pleophylla sp 1	834710	L1	KC904117	KC964448	KC964229	KC964339
Pleophylla sp 1	834711	L1	KC904118	KC964449	KC964230	KC964340
Pleophylla sp 1	834712*	L1	KC904119	KC964450	KC964231	KC964341
Pleophylla sp 1	834713	L1	KC904120	KC964451	KC964232	KC964342
Pleophylla sp 1	834714	L1	KC904121	KC964452	KC964233	KC964343
Pleophylla sp 1	834715*	L1	KC904122	KC964453	KC964234	KC964344
Pleophylla sp 1	834717*	L1	KC904124	KC964455	KC964236	KC964346
Pleophylla sp 1	834718	L1	KC904125	KC964456	KC964237	KC964347
Pleophylla sp 1	834826*	L1	KC904195	KC964523	KC964307	KC964416
Pleophylla sp 1	834827	L1	KC904196	KC964524	KC964308	KC964417
Pleophylla sp 1	834828*	L1	KC904197	KC964525	KC964309	KC964418
Pleophylla sp 10	834723*	L2	KC904130	KC964461	KC964242	NA
Pleophylla sp 10	834724*	L2	KC904131	KC964462	KC964243	KC964352
Pleophylla sp 10	834725*	L2	KC904132	KC964463	KC964244	KC964353
Pleophylla sp 10	834736*	L3	KC904143	KC964474	KC964255	KC964364
Pleophylla sp 10	834739*	L3	KC904146	KC964477	KC964258	KC964367
Pleophylla sp 10	834742	L3	KC904149	KC964480	KC964261	KC964370
Pleophylla sp 10	834743*	L3	KC904150	KC964481	KC964262	KC964371
Pleophylla sp 10	834745*	L3	KC904152	KC964483	KC964264	KC964373
Pleophylla sp 10	834746	L3	KC904153	KC964484	KC964265	KC964374
Pleophylla sp 10	834747	L3	KC904154	KC964485	KC964266	KC964375
Pleophylla sp 10	834748*	L3	KC904155	KC964486	KC964267	KC964376
Pleophylla sp 10	834749	L3	KC904156	KC964487	KC964268	KC964377
Pleophylla sp 10	834750*	L6	KC904157	KC964488	KC964269	KC964378
Pleophylla sp 10	834751	L6	KC904158	NA	KC964270	KC964379
Pleophylla sp 10	834752	L6	KC904159	KC964489	KC964271	KC964380
Pleophylla sp 10	834757*	L3	KC904164	KC964494	KC964276	KC964385
Pleophylla sp 10	834758*	L3	KC904165	KC964495	KC964277	KC964386
Pleophylla sp 10	834759*	L3	KC904166	KC964496	KC964278	KC964387
Pleophylla sp 10	834760*	L3	KC904167	KC964497	KC964279	KC964388
Pleophylla sp 10	834761	L3	KC904168	KC964498	KC964280	KC964389
Pleophylla sp 10	834762	L3	KC904169	KC964499	KC964281	KC964390
Pleophylla sp 10	834763*	L3	KC904170	KC964500	KC964282	KC964391
Pleophylla sp 10	834764*	L3	KC904171	KC964501	KC964283	KC964392
Pleophylla sp 10	834765*	L3	KC904172	KC964502	KC964284	KC964393
Pleophylla sp 10	834766*	L3	KC904173	KC964503	KC964285	KC964394
Pleophylla sp 10	834767*	L3	KC904174	KC964504	KC964286	KC964395
Pleophylla sp 10	834768*	L3	KC904175	KC964505	KC964287	KC964396
Pleophylla sp 10	834769	L3	KC904176	KC964506	KC964288	KC964397
Pleophylla sp 10	834770*	L3	KC904177	KC964507	KC964289	KC964398
Pleophylla sp 10	834771*	L3	KC904178	KC964508	KC964290	KC964399
Pleophylla sp 10	834772*	L3	KC904179	KC964509	KC964291	KC964400
Pleophylla sp 10	834834*	L2	KC904200	KC964528	KC964312	KC964421
Pleophylla sp 10	834924*	L3	KC904202	KC964530	KC964314	KC964423
Pleophylla sp 10	835048*	L6	KC904207	KC964535	KC964319	KC964428
Pleophylla sp 11	834719*	L2	KC904126	KC964457	KC964238	KC964348
Pleophylla sp 11	834733*	L4	KC904140	KC964471	KC964252	KC964361
Pleophylla sp 11	834737*	L3	KC904144	KC964475	KC964256	KC964365
Pleophylla sp 11	834738	L3	KC904145	KC964476	KC964257	KC964366
Pleophylla sp 11	834740*	L3	KC904147	KC964478	KC964259	KC964368
Pleophylla sp 11	834741*	L3	KC904148	KC964479	KC964260	KC964369
Pleophylla sp 11	834744*	L3	KC904151	KC964482	KC964263	KC964372
Pleophylla sp 11	834773*	L3	KC904180	KC964510	KC964292	KC964401
Pleophylla sp 11	834774*	L3	KC904181	KC964511	KC964293	KC964402
Pleophylla sp 11	834775*	L3	KC904182	KC964512	KC964294	KC964403
Pleophylla sp 11	834776*	L3	KC904183	NA	KC964295	KC964404

Supplementary Table S1 (cont.)

Pleophylla sp 11	834777*	L3	KC904184	KC964513	KC964296	KC964405
Pleophylla sp 11	834778*	L3	KC904185	KC964514	KC964297	KC964406
Pleophylla sp 11	834779	L3	KC904186	KC964515	KC964298	KC964407
Pleophylla sp 11	834780	L3	KC904187	KC964516	KC964299	KC964408
Pleophylla sp 11	834781	L3	KC904188	KC964517	KC964300	KC964409
Pleophylla sp 11	834782	L3	KC904189	KC964518	KC964301	KC964410
Pleophylla sp 11	834783	L3	KC904190	KC964519	KC964302	KC964411
Pleophylla sp 11	834784	L3	KC904191	KC964520	KC964303	KC964412
Pleophylla sp 11	834785*	L3	KC904192	KC964521	KC964304	KC964413
Pleophylla sp 11	834786	L3	KC904193	KC964522	KC964305	KC964414
Pleophylla sp 11	834832*	L2	KC904198	KC964526	KC964310	KC964419
Pleophylla sp 11	834925*	L3	KC904203	KC964531	KC964315	KC964424
Pleophylla sp 12	834926*	L3	KC904204	KC964532	KC964316	KC964425
Pleophylla sp 2	834693*	L1	KC904100	KC964431	KC964212	KC964322
Pleophylla sp 2	834695	L1	KC904102	KC964433	KC964214	KC964324
Pleophylla sp 2	834702*	L1	KC904109	KC964440	KC964221	KC964331
Pleophylla sp 2	834705*	L1	KC904112	KC964443	KC964224	KC964334
Pleophylla sp 2	834707*	L1	KC904114	KC964445	KC964226	KC964336
Pleophylla sp 2	834716*	L1	KC904123	KC964454	KC964235	KC964345
Pleophylla sp 2	834825*	L1	KC904194	NA	KC964306	KC964415
Pleophylla sp 6	834720*	L2	KC904127	KC964458	KC964239	KC964349
Pleophylla sp 6	834721*	L2	KC904128	KC964459	KC964240	KC964350
Pleophylla sp 6	834722*	L2	KC904129	KC964460	KC964241	KC964351
Pleophylla sp 6	834726*	L2	KC904133	KC964464	KC964245	KC964354
Pleophylla sp 6	834727	L2	KC904134	KC964465	KC964246	KC964355
Pleophylla sp 6	834833*	L2	KC904199	KC964527	KC964311	KC964420
Pleophylla sp 6	835042*	L8	KC904205	KC964533	KC964317	KC964426
Pleophylla sp 9	834734	L4	KC904141	KC964472	KC964253	KC964362
Pleophylla sp 9	834735*	L4	KC904142	KC964473	KC964254	KC964363
Pleophylla sp 9	834904*	L4	KC904201	KC964529	KC964313	KC964422
Pleophylla sp X2	835047*	L6	KC904206	KC964534	KC964318	KC964427
Pleophylla sp (indet.)	834696	L1	KC904103	KC964434	KC964215	KC964325
Pleophylla sp (indet.)	834697	L1	KC904104	KC964435	KC964216	KC964326
Pleophylla sp (indet.)	834698	L1	KC904105	KC964436	KC964217	KC964327
Pleophylla sp (indet.)	834728	L5	KC904135	KC964466	KC964247	KC964356
Pleophylla sp (indet.)	834729	L5	KC904136	KC964467	KC964248	KC964357
Pleophylla sp (indet.)	834730	L5	KC904137	KC964468	KC964249	KC964358
Pleophylla sp (indet.)	834731	L5	KC904138	KC964469	KC964250	KC964359
Pleophylla sp (indet.)	834732	L5	KC904139	KC964470	KC964251	KC964360
Pleophylla sp (indet.)	834753	L7	KC904160	KC964490	KC964272	KC964381
Pleophylla sp (indet.)	834754	L7	KC904161	KC964491	KC964273	KC964382
Pleophylla sp (indet.)	834755	L7	KC904162	KC964492	KC964274	KC964383
Pleophylla sp (indet.)	834756	L7	KC904163	KC964493	KC964275	KC964384

Table S2. Collection localities with their geographical coordinates.

ID	Name	Lat	Long
**L1**	South Africa: Cheerio Farm, Haenertsburg, ca. 20 km W of Tzaneen, 1492 m	-23.895	29.953
**L2**	South Africa: Drakensberge, Highmoor, 1582 m, at light	-29.331	29.691
**L3**	South Africa: Kwazulu-Natal: Drakensberge Lodge 15 km NW of Himeville, 1622 m, at light	-29.631	29.419
**L4**	South Africa: Limpopo: Legalameetse Nature Reserve, guest house camp (Murchson range), 814 m, at light	-24.200	30.337
**L5**	South Africa: Kwazulu-Natal: Nature Farm (Louwsburg), 1100 m, at light	-27.567	31.300
**L6**	South Africa: Free State: Wakkerstrom (Wetland Lodge env.), 1802 m	-27.339	30.153
**L7**	South Africa: Eastern Cape: Morgans Bay env. (Yellowwood Tree Park), 4 m	-32.696	28.334
**L8**	South Africa: Fort Fordyce, 1000 m	-32.410	26.280

Table S3. Optimal partition schemes and substitution models selected for each phylogenetic program using PartitionFinder (Lanfear et al., 2012) under the Bayesian Information Criterion (BIC). Note that when invariant (I) and gamma distributed (Γ) sites both featured in the optimal model, we implemented only the Γ parameter (see materials and method for details).

Dataset	Package	Partition scheme (model)
cox1	PhyML	Unpartitioned (HKY + I + Γ)
	RAxML	Codon 1 + 2 (GTR + Γ), Codon 3 (GTR + Γ)
	MrBayes	Codon 1 (GTR + I), Codon 2 (F80 + I), Codon 3 (GTR + Γ)
MtDNA	PhyML	Unpartitioned (HKY + Γ)
	RAxML	Codon 1 + 2 (GTR + Γ), Codon 3 (GTR + Γ), 16S (GTR + Γ)
	MrBayes	Codon 1 (GTR + I), Codon 2 (F80 + I), Codon 3 (GTR + Γ), 16S (HKY + Γ)
NucDNA	PhyML	Unpartitioned (K80 + Γ)
	RAxML	ITS1 (GTR + I + Γ), 28S (GTR + Γ)
	MrBayes	ITS1 (K80 + Γ), 28S (K80 +I)
Mt+Nuc	PhyML	Unpartitioned (HKY + I + Γ)
	RAxML	Codon 1 (GTR + Γ), Codon 2 (GTR + Γ), Codon 3 (GTR + Γ), 16S (GTR + Γ), ITS1 (GTR + I + Γ), 28S (GTR + Γ)
	MrBayes	Codon 1 (GTR + Γ), Codon 2 (F81), Codon 3 (GTR + Γ), 16S (HKY + Γ), ITS1 (K80 + Γ), 28S (K80 + I)

Table S4. Maximum likelihood estimates of tree length obtained for each marker using maximum likelihood phylogenetic analysis (PhyML and RAxML) and Bayesian phylogenetic analysis under different branch length priors (MrBayes). Posterior means and intervals are presented for the Bayesian estimates.

Analysis	MLE[a]	Mean (95% HPDs)[b]	MLE	Mean (95% HPDs)	MLE	Mean (95% HPDs)	MLE	Mean (95% HPDs)
	cox1		*rrnL*[u]		mtDNA			
MrBayes *Exp*(10)[c]	14.98	17.12 (14.25, 19.99)	23.2	21.85 (19.13, 24.84)	23.41	21.77 (19.12, 24.67)		
MrBayes *Exp* (20)	6.47	7.34 (6.18, 8.58)	10	10.9 (9.39, 12.3)	9.91	10.93 (9.59, 12.41)		
MrBayes *Exp*(100)	1.54	1.43 (1.25, 1.61)	1.45	1.82 (1.49, 2.15)	1.17	1.25 (1.09, 1.41)		
MrBayes *Exp*(200)	1.051	1 (0.9, 1.1)	0.65	0.77 (0.65, 0.89)	0.9	0.88 (0.79, 0.96)		
PhyML	1.43[u]	-	0.28	-	1.14[u]	-		
RAxML	2.12	-	0.27	-	1.38	-		
	ITS1[u]		28S[u]		nucDNA		*Combined matrix*	
MrBayes *Exp*(10)	19.31	21.33 (18.48, 24.43)	6.7	10.22 (8.2, 12.36)	19.9	21.8 (18.88, 24.84)	NA[d]	NA
MrBayes *Exp*(20)	8.99	10.47 (9.13, 11.95)	3.95	5.48 (4.53, 6.4)	9.61	10.76 (9.35, 10.24)	10.7	10.7 (9.29, 12.1)
MrBayes *Exp*(100)	0.7	0.84 (0.69, 0.98)	1.19	1.56 (1.34, 1.78)	0.93	1.59 (1.13, 1.13)	1.14	1.14 (0.96, 1.33)
MrBayes *Exp*(200)	0.55	0.59 (0.52, 0.66)	0.71	0.86 (0.74, 0.98)	0.44	0.75 (0.63, 0.79)	0.78	0.78 (0.7, 0.86)
PhyML	0.4	-	0.01	-	0.23[u]	-	0.69[u]	-
RAxML	0.4	-	0.01	-	0.27	-	1.05	-

a Maximum likelihood estimate of tree length (sum of branch lengths)
b Posterior means and 95% highest posterior density intervals for tree length
c Exponential prior distribution *Exp*(λ), where λ is the rate parameter
d NA = analysis failed to converge
u Unpartitioned analysis

Table S5. The estimated mean rates (± SD) of *cox1* partitions and ITS1 estimated using *BEAST, relative to the fixed rate of *cox1*.

cox1 ucld.mean [a]	*cox1* mean rate [b]	*cox1* : 1st codon [c]	*cox1* : 2nd codon	*cox1* : 3rd codon	ITS1 ucld.mean	ITS1 mean rate
0.01 (2%) [d]	0.0094 ± 0.001	0.0011 ± 0.032	0.0004 ± 0.019	0.0267 ± 0.039	0.0017 ± 0.001	0.0024 ± 0.001
0.0125 (2.5%)	0.0118 ± 0.002	0.0014 ± 0.032	0.0005 ± 0.019	0.0336 ± 0.039	0.002 ± 0.001	0.0029 ± 0.001
0.015 (3%)	0.014 ± 0.002	0.0016 ± 0.033	0.0006 ± 0.019	0.04 ± 0.04	0.0029 ± 0.011	0.0035 ± 0.004
0.0175 (3.5%)	0.0165 ± 0.002	0.0019 ± 0.032	0.0007 ± 0.019	0.0477 ± 0.039	0.0029 ± 0.001	0.0042 ± 0.002
0.02 (4%)	0.019 ± 0.002	0.0022 ± 0.033	0.0008 ± 0.019	0.0541 ± 0.039	0.0033 ± 0.001	0.0047 ± 0.002

a Mean of the uncorrelated lognormal relaxed clock, equivalent to the mean branch rate

b Mean rate of evolution across the whole tree

c The rate of evolution for individual codons is obtained by multiplying the mean rate of *cox1* by the relative codon rate

d Percentage divergence My^{-1}

Table S6. Interspecific divergence times estimated using *BEAST under variable *cox1* substitution rates.

Split	2% My^{-1} Mean (95% HPDs)	2.5% My^{-1} Mean (95% HPDs)	3% My^{-1} Mean (95% HPDs)	3.5% My^{-1} Mean (95% HPDs)	4% My^{-1} Mean (95% HPDs)
01 + 02	0.39 (0.01, 0.17)	0.07 (0.01, 0.14)	0.06 (0.01, 0.12)	0.05 (0, 0.1)	0.04 (0, 0.09)
06 + 10	0.09 (0.17, 0.65)	0.31 (0.14, 0.51)	0.26 (0.11, 0.43)	0.23 (0.1, 0.37)	0.19 (0.09, 0.32)
01 + 02, 06 + 10	3.17 (1.51, 4.82)	2.52 (1.22, 3.82)	2.11 (1.02, 3.24)	1.81 (0.85, 2.73)	1.56 (0.77, 2.34)
01 + 02 + 06 + 10, 11	17.53 (7.17, 34.73)	13.48 (5.33, 26.12)	11.73 (1.75, 21.04)	9.77 (4.05, 18.87)	8.32 (3.64, 15.09)
01 + 02 + 06 + 10 + 11, 9 (root)	20.23 (2.64, 35.97)	15.56 (1.96, 26.77)	13.42 (1.58, 24.43)	11.26 (1.56, 19.84)	9.55 (3.69, 17.88)

Table S7. Results of the JML analysis based on *cox1* codon partitions [P1/P2/P3] and ITS1: minimum distances (below the diagonal) and *P*-values (above the diagonal, shaded in grey). Values are presented for trees obtained using the *cox1* rate 2% My^{-1}, but note the results are equivalent for trees generated under different substitution rates.

		Species 01	*Species 02*	*Species 06*	*Species 09*	*Species 10*	*Species 11*
cox1	*Species 01*	-	1 / 1 / 0.66	0.43 / 0.49 / 0.16	0.1 / 0.26/ **0.03**	0.17 / 0.49 / 0.21	0.3 / 0.14 / **0.03**
	Species 02	0 / 0 / 0	-	0.42 / 0.48 / 0.14	0.17 / 0.43 / **0.03**	0.17 / 0.48/ 0.19	0.29 / 0.3 / **0.04**
	Species 06	0 / 0 / 0.11	0 / 0 / 0.11	-	0.24 / 0.43 / **0.03**	0.75 / 0.89 / 0.26	0.2 / 0.3 / **0.04**
	Species 09	0.02 / 0.01 / 0.29	0.02 / 0.01 / 0.29	0.03 / 0.01 / 0.29	-	0.17 / 0.46 / **0.03**	0.42 / 0.42 / **0.03**
	Species 10	0 / 0 / 0.11	0 / 0 / 0.11	0 / 0 / 0.01	0.02 / 0.01 / 0.29	-	0.2 / 0.29 / **0.04**
	Species 11	0.03 / 0 / 0.3	0.03 / 0.01 / 0.3	0.02 / 0.01 / 0.29	0.03 / 0.01 / 0.28	0.02 / 0.01 / 0.29	-
ITS1							
	Species 01	-	0.97	0.29	0.89	0.92	0.76
	Species 02	0	-	0.26	0.89	0.92	0.75
	Species 06	0.01	0.01	-	0.88	0.99	0.48
	Species 09	0.12	0.12	0.12	-	0.9	0.99
	Species 10	0.04	0.04	0.02	0.12	-	0.78
	Species 11	0.09	0.09	0.08	0.12	0.09	-

Table S8. Results of the of the GMYC model for species delineation and statistical parsimony analysis based on *cox1* and ITS1.

	Tree[a]	$logL_{Null}$[b]	$logL_{GMYC-Single}$[c]	$logL_{GMYC-Multi}$[d]	AIC_c[e]	Akaike weight[f]	Entities[g]	Variance (σ^2)[h]
cox1								
	MrBayes *Exp*(10)[i] + PATHd8	285.49	293.61***[j]	293.75	-575.93	0.22	15.12 (3)[k]	0.92
	MrBayes *Exp*(20) + PATHd8	283.39	288.93*	289.77	-566.59	0.17	16.43 (4)	0.89
	MrBayes *Exp*(100) + PATHd8	264.44	267.84	526.59***	-1039.35	1	3.00 (1)	0.00
	MrBayes *Exp*(200) + PATHd8	258.45	265.51	268.17***	-522.52	0.54	15.00 (1)	0.00
	RAxML + PATHd8	321.56	386.43	840.18***	-1663.87	0.5	4.00 (2)	0.00
	MrBayes *Exp*(10) + R8S	268.04	278.24	280.61***	-547.48	0.22	16.33 (2)	0.44
	MrBayes *Exp*(20) + R8S	260.21	269.56***	270.29	-527.84	0.15	11.92 (7)	1.54
	MrBayes *Exp*(100) + R8S	245.94	253.77	255.10**	-496.38	0.13	12.73 (8)	2.36
	MrBayes *Exp*(200) + R8S	239.96	245.02	247.89**	-481.95	0.37	12.00 (1)	0.00
	RAxML + R8S	283.23	290.25**	290.71	-569.22	0.23	16.54 (3)	0.89
	Statistical Parsimony	-	-	-	-		13.00	-
ITS1								
	MrBayes *Exp*(10) + PATHd8	70.02	75.70**	75.76	-137.41	0.34	10.99 (4)	4.05
	MrBayes *Exp*(20) + PATHd8	69.85	77.29**	77.29	-140.57	0.7	8.00 (1)	0.00
	MrBayes *Exp*(100) + PATHd8	67.84	74.45**	74.45	-134.91	0.59	8.00 (1)	0.00
	MrBayes *Exp*(200) + PATHd8	65.89	72.08**	72.08	-130.16	0.43	8.00 (1)	0.00
	RAxML + PATHd8	70.50	77.33**	77.49	-140.66	0.6	8.00 (1)	0.00
	MrBayes *Exp*(10) + R8S	65.95	72.02**	72.02	-130.05	0.49	8.00(1)	0.00
	MrBayes *Exp*(20) + R8S	65.60	71.60**	71.60	-129.21	0.48	8.00 (1)	0.00
	MrBayes *Exp*(100) + R8S	63.45	69.02*	69.02	-124.04	0.43	9.34 (3)	5.22
	MrBayes *Exp*(200) + R8S	64.70	71.16**	71.35	-128.32	0.58	8.00 (1)	0.00
	RAxML + R8S	71.36	79.76***	79.76	-145.52	0.75	8.00 (1)	0.00
	Statistical Parsimony	-	-	-	-		9	-
rrnL								
	Statistical Parsimony	-	-	-	-		1	-
28S								
	Statistical Parsimony	-	-	-	-		1	-

a Tree building and (+) tree linearization method, with the exception of statistical parsimony
b The likelihood of the null model
c The likelihood of the GMYC single threshold model
d The likelihood of the GMYC multiple threshold model
e The AIC_c score of the preferred model
f Akaike weight of the preferred model
g Model averaged entities within $\delta AIC_c = 2$
h Variance in the total number of entities
i Exponential prior distribution *Exp*(λ), where λ is the rate parameter
j Preferred model, likelihood ratio test ***$P < 0.001$, **$P < 0.01$, *$P < 0.05$
k Number of models within $\delta AIC_c = 2$

Table S9. Single gene analysis output.

- ➔ **Excel file 'Table_S9.xls'**

Table S10. Results of the Eigenshape analysis: eigenvalues, total variance and total cumulative variation expressed by the first fourteen Eigenshapes.

Eigenshape	Eigenvalue	Total variance (%)	Cumulative variance (%)
ES1	23.93	51.48	51.48
ES2	7.25	15.6	67.08
ES3	3.15	6.78	73.86
ES4	2.79	6.01	79.87
ES5	1.79	3.86	83.73
ES6	1.28	2.75	86.48
ES7	1.07	2.31	88.79
ES8	0.78	1.69	90.48
ES9	0.49	1.06	91.54
ES10	0.46	0.98	92.52
ES11	0.39	0.84	93.36
ES12	0.38	0.81	94.17
ES13	0.35	0.75	94.92
ES14	0.25	0.54	95.46

Table S11. Results of LDA reflecting the fit of morphometric data to *a priori* defined morphospecies showing for each species sample size (n) and percentage of correctly reassigned individuals.

		% corr.	
**Species**	**n**	**ES 1-4**	**ES 1-14**
**total**	68	84.85	89.39
**sp1**	12	91.67	91.67
**sp2**	6	33.33	50
**sp6**	5	60	80
**sp9**	2	0	0
**sp10**	26	96.15	100
**sp11**	15	100	100
**sp12**	1	0	0
**spX2**	1	0	0

Table S12. Results of LDA reflecting the fit of morphometric data to the groups recognized with cluster analysis showing for each species sample size (n) and percentage of correctly reassigned individuals.

	ES 1-4		ES 1-14	
**Cluster**	**n**	**% corr.**	**n**	**% corr.**
**total**	68	100	68	94.12
**Cluster 1**	28	100	6	100
**Cluster 2**	25	100	2	100
**Cluster 3**	15	100	6	100
**Cluster 4**	-	-	10	70
**Cluster 5**	-	-	8	100
**Cluster 6**	-	-	3	100
**Cluster 7**	-	-	5	100
**Cluster 8**	-	-	6	83.33
**Cluster 9**	-	-	3	100
**Cluster 10**	-	-	11	100
**Cluster 11**	-	-	4	100
**Cluster 12**	-	-	4	100

Table S13. Posterior probabilities of speciation splits of all guide trees for prior only analyses and 3 data sets, 9 τ and θ prior combinations, and 10 repeats of each analysis.

- ➔ **Excel file 'Table_S12.xls'**

Table S14. Lumping behaviour of BPP analyses with simultaneous species tree estimation. Posterior probabilities for species delimitation scenarios are shown for all initial guide trees and all θ and τ_0 prior combinations. Lumped species are indicated by parentheses. Scenarios with pp < 0.05 in all analyses are not shown.

- ➔ **Excel file 'Table_S13.xls'**

Table S15. Posterior probabilities for a priori defined morphospecies from BPP analyses with simultaneous species tree estimation for all initial guide trees. Species that were never sampled are denoted with 'na'.

- ➔ **Excel file 'Table_S14.xls'**

Figure S1. Plots of axes 1 and 2 from canonical variate analysis of the left paramere of male *Pleophylla* specimens. Specimens are colored according to morphospecies assignments (Table S1). The digitized outline of the left paramere is indicated in blue line for sp11 (see arrow).

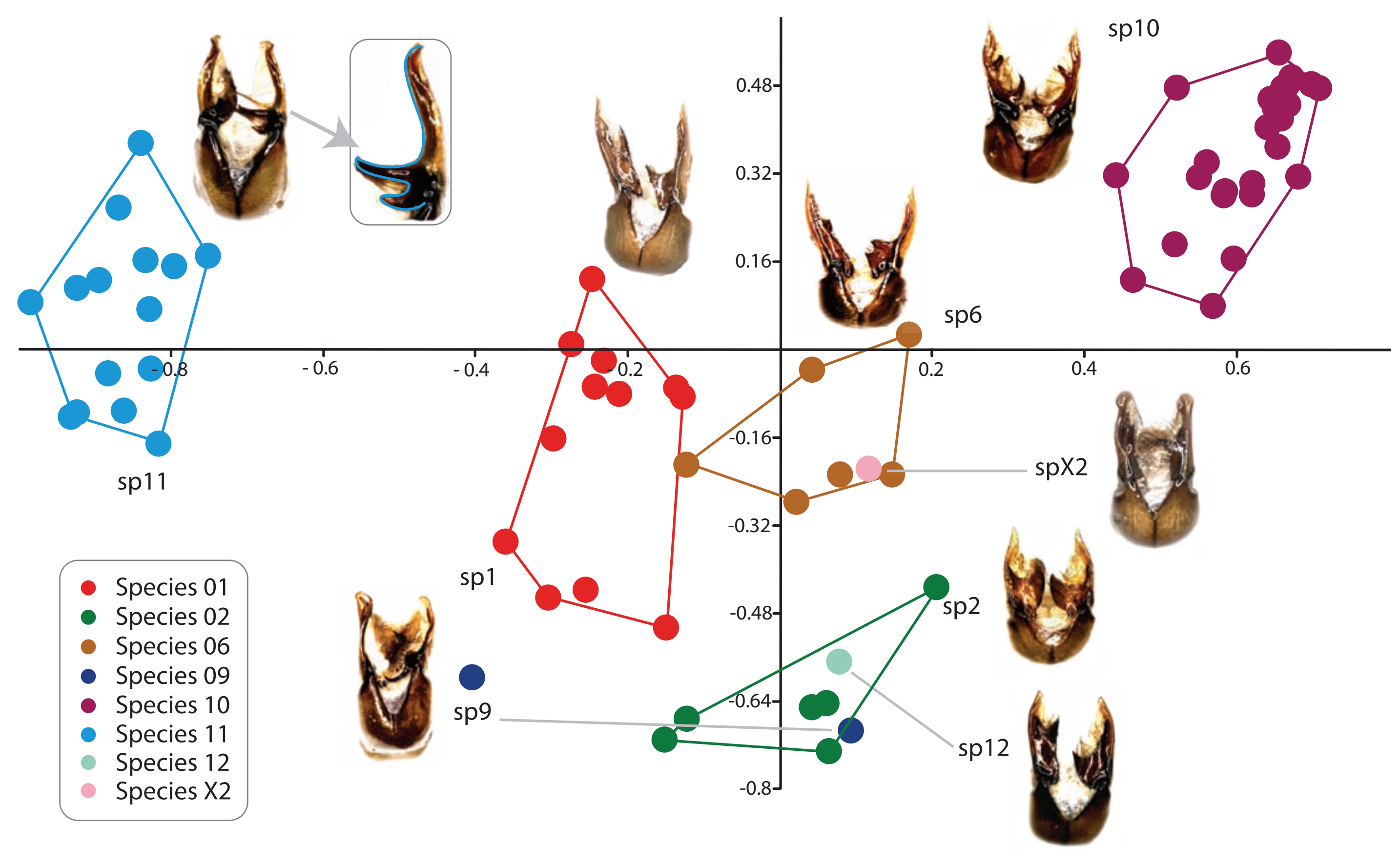

sp10
sp6
sp11
spX2
sp1
sp2
sp9
sp12
0.48
0.32
0.16
-0.16
-0.32
-0.48
-0.64
-0.8
- 0.8
- 0.6
- 0.4
- 0.2
0.2
0.4
0.6
Species 01
Species 02
Species 06
Species 09
Species 10
Species 11
Species 12
Species X2

Figure S2. Guide tree topologies for BPP analyses and speciation split labelling. (A) Tree A: *BEAST topology, sp12 and spX2 were inserted according to the RAxML topology, (B) RAxML and MrBayes topology, and (C) topology derived from morphological similarity. Identical splits are labelled with the same number.

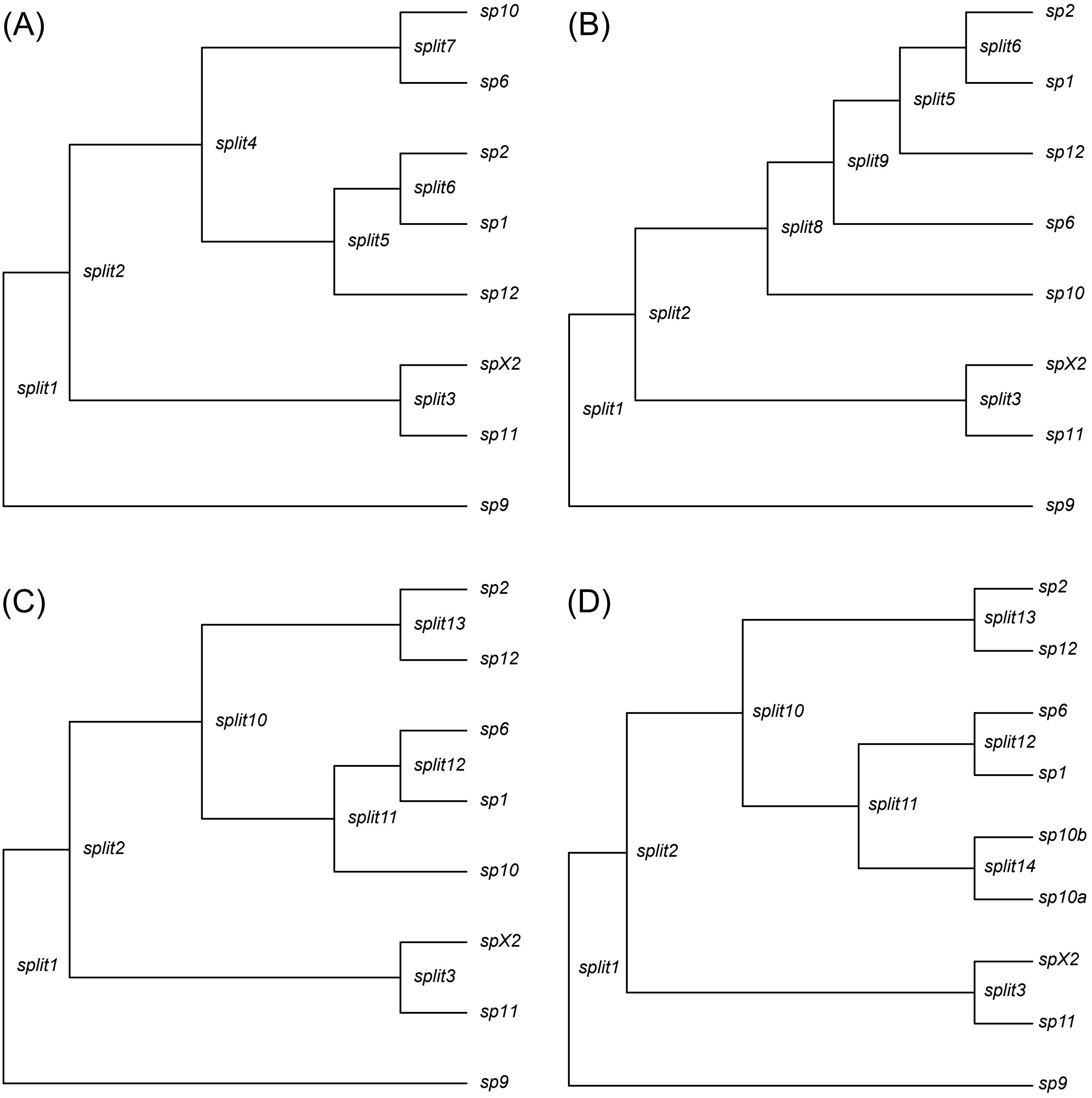
(A)
sp10
split7
sp6
split4
sp2
split6
sp1
split5
split2
sp12
spX2
split1
split3
sp11
sp9
(B)
sp2
split6
sp1
split5
sp12
split9
sp6
split8
sp10
split2
spX2
split1
split3
sp11
sp9
(C)
sp2
split13
sp12
split10
sp6
split12
sp1
split11
split2
sp10
spX2
split1
split3
sp11
sp9
(D)
sp2
split13
sp12
split10
sp6
split12
sp1
split11
sp10b
split2
split14
sp10a
spX2
split1
split3
sp11
sp9

Figure S3. Maximum likelihood (RAxML) trees of *Pleophylla* for independent and combined molecular datasets (mt: mitochondrial, n: nuclear). Specimens are colored according to morphospecies assignments (Table S1). Branch length corresponds to inferred numbers of substitutions per site. Bootstrap support values are indicated for interspecific divergences. Values less than 50% are not shown.

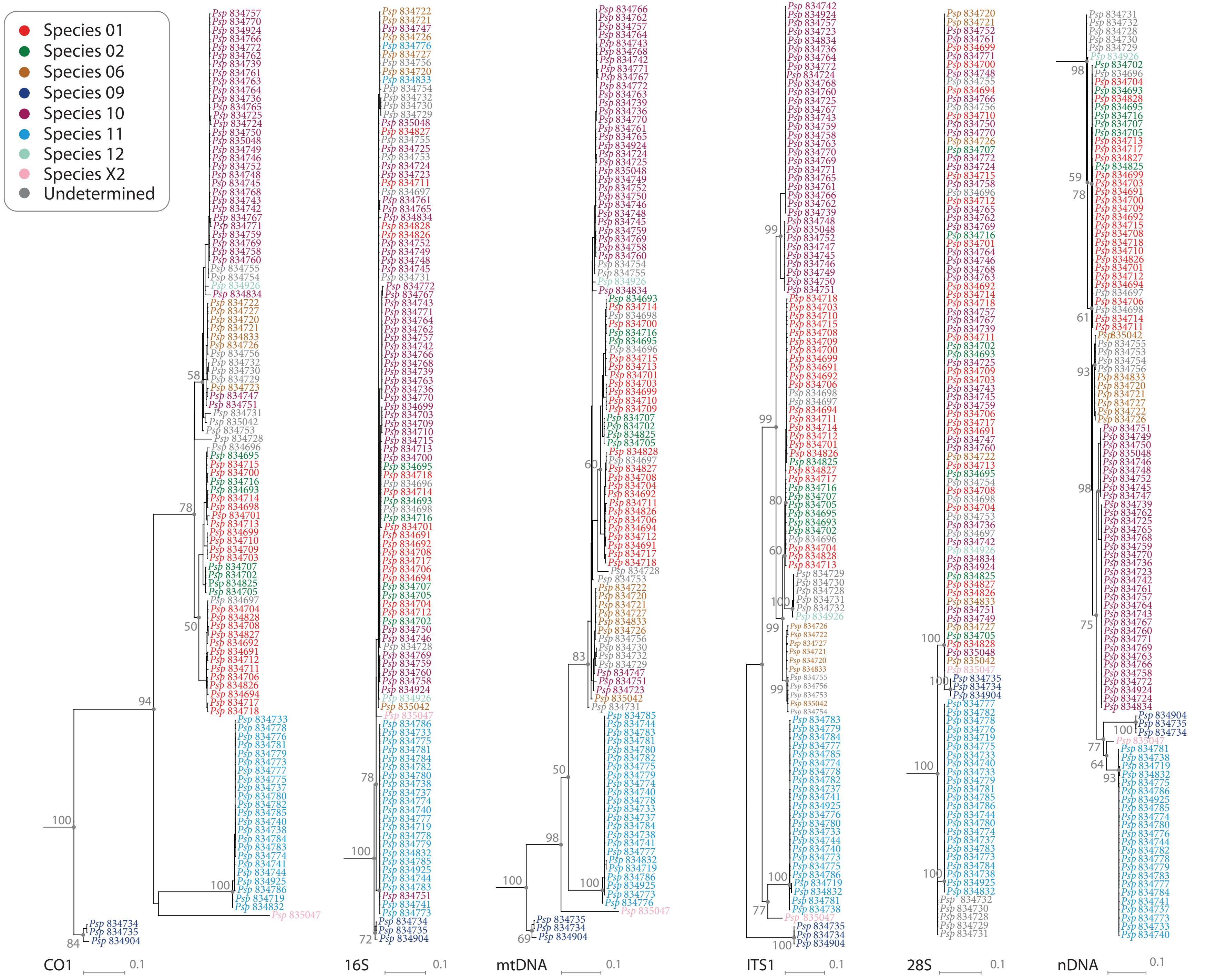
Species 01
Species 02
Species 06
Species 09
Species 10
Species 11
Species 12
Species X2
Undetermined
CO1
16S
mtDNA
ITS1
28S
nDNA
0.1

Figure S4. Maximum likelihood (PhyML) trees of *Pleophylla* (Psp) for independent and combined molecular datasets. Specimens are colored according to morphospecies assignments (Table S1). Branch length corresponds to inferred numbers of substitutions per site. Bayes support values are indicated for interspecific divergences. Values less than 0.5 are not shown.

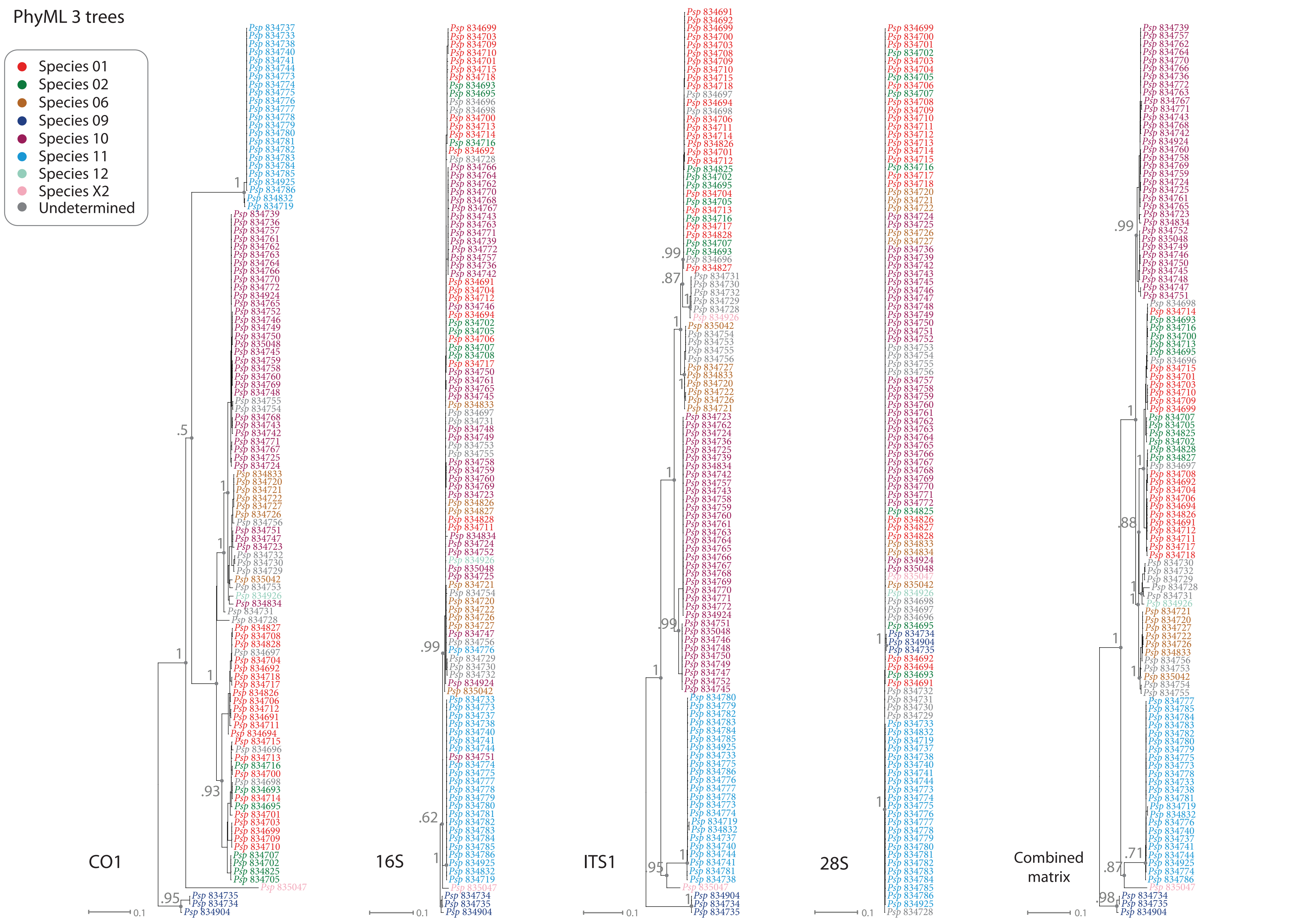

PhyML 3 trees
Species 01
Species 02
Species 06
Species 09
Species 10
Species 11
Species 12
Species X2
Undetermined
CO1
16S
ITS1
28S
Combined matrix
0.1

Figure S5. Bayesian (MrBayes) trees of *Pleophylla* (Psp) for independent and combined molecular datasets. Specimens are colored according to morphospecies assignments (Table S1). Branch length corresponds to inferred numbers of substitutions per site. Bayesian posterior probabilities are indicated for interspecific divergences. Values less than 0.5 are not shown.

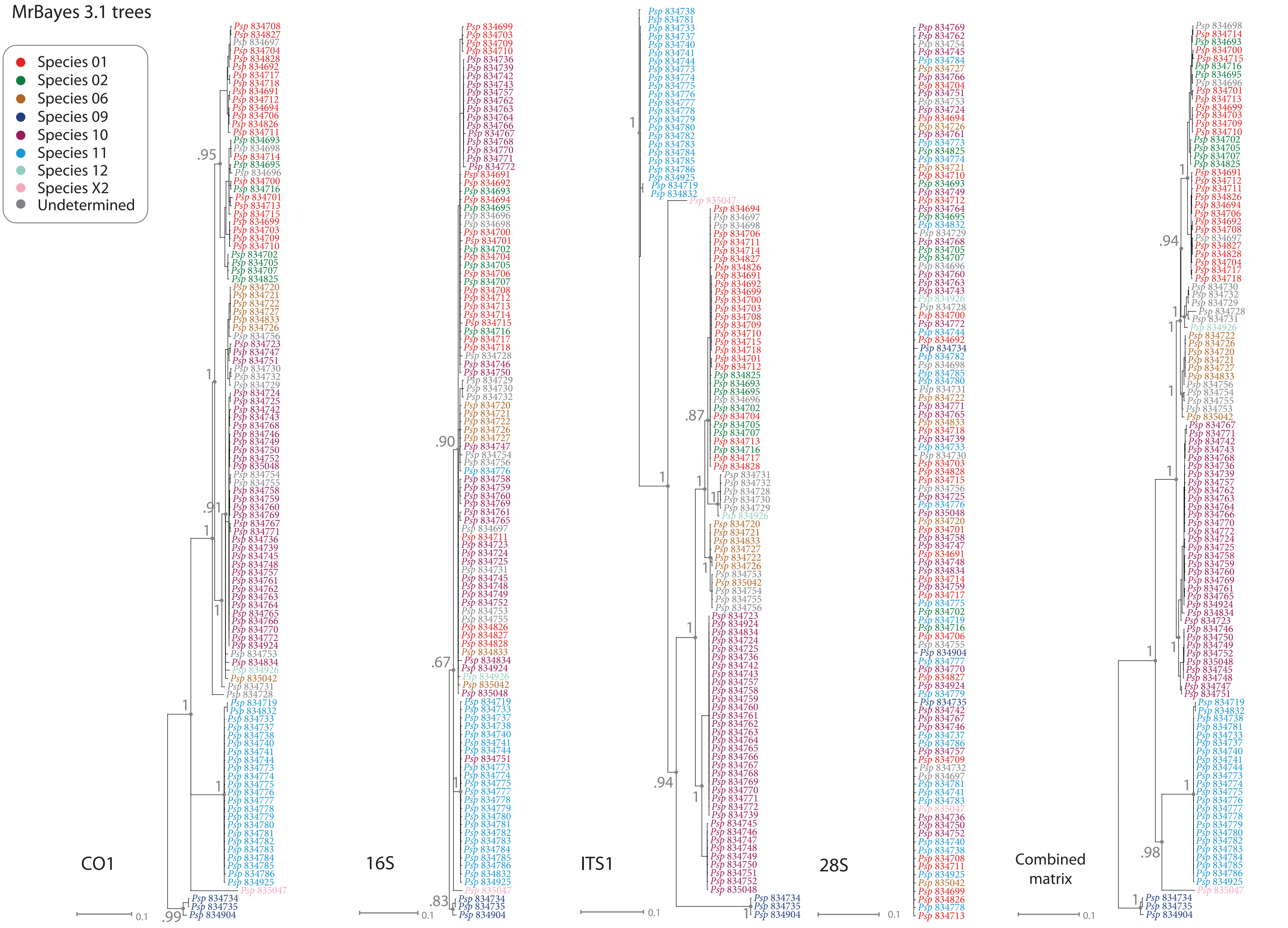

MrBayes 3.1 trees
Species 01
Species 02
Species 06
Species 09
Species 10
Species 11
Species 12
Species X2
Undetermined
CO1
16S
ITS1
28S
Combined matrix
0.1

Figure S6. Pairwise plots of Eigenshape axes 1-3 from the Eigenshape analysis of partial paramere outlines.

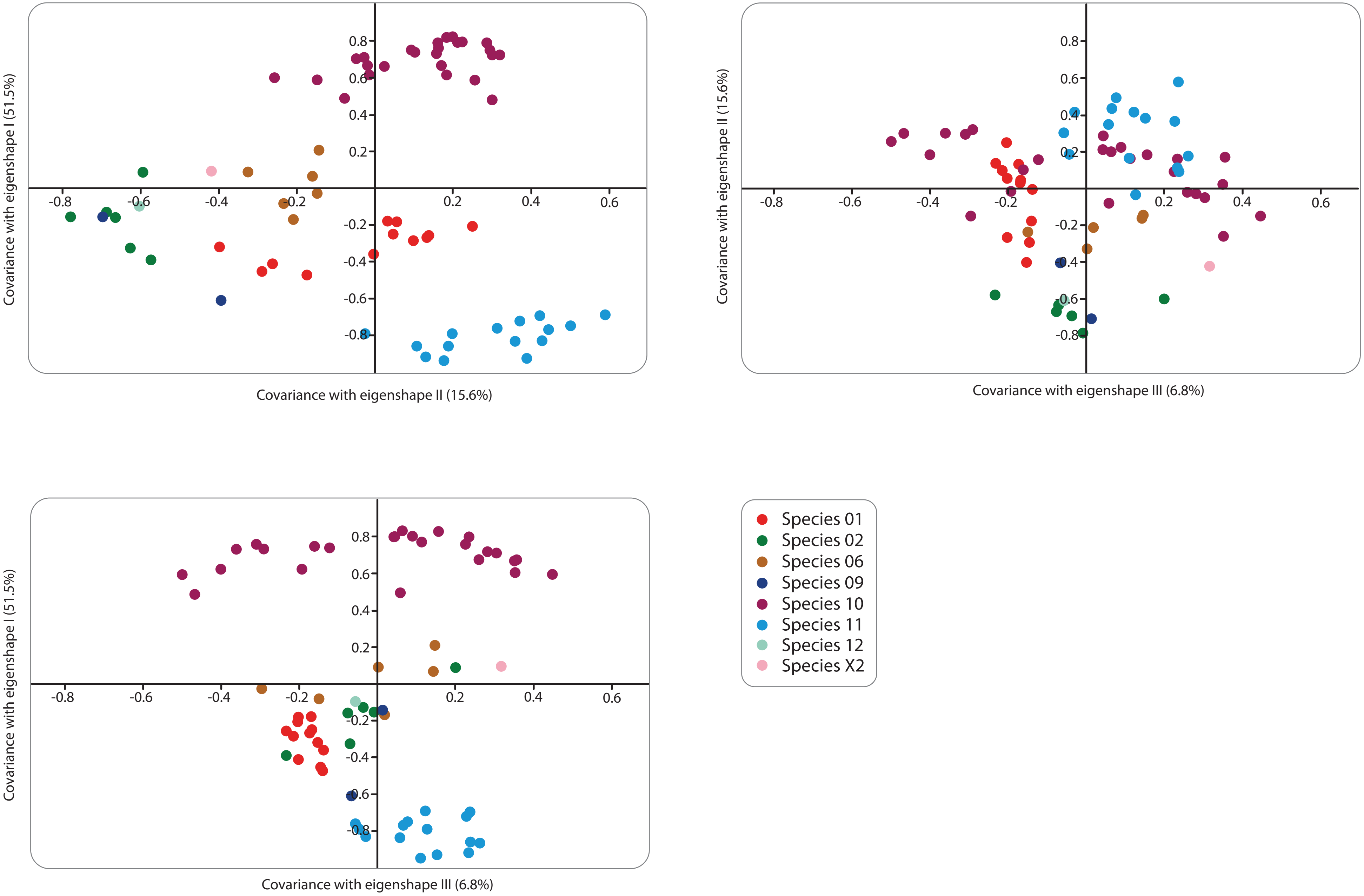
Covariance with eigenshape I (51.5%)
Covariance with eigenshape II (15.6%)
Covariance with eigenshape II (15.6%)
Covariance with eigenshape III (6.8%)
Covariance with eigenshape I (51.5%)
Covariance with eigenshape III (6.8%)
-0.8
-0.6
-0.4
-0.2
0.2
0.4
0.6
0.8
Species 01
Species 02
Species 06
Species 09
Species 10
Species 11
Species 12
Species X2

Figure S7. Mean posterior probabilities of Bayesian species delimitations from 10 repeated runs with commonly used priors using the additional guide tree. Means inferred under 9 different θ and τ prior combinations are color-coded in 3x3 boxes on each putative speciation split of the guide trees. The arrows in the legend point to the direction of more conservative prior choices. Results are shown for analyses using (A) no data (prior only), (B) molecular data, (C) morphometric trait data, and (D) both data sources. The colours of the large 3x3 inset boxes indicate the number of repeat-analyses that were stuck in the one species model. Gamma distribution densities of θ and τ priors 1-3 are depicted in the bottom left corner. Dashed lines indicate the respective distribution means.

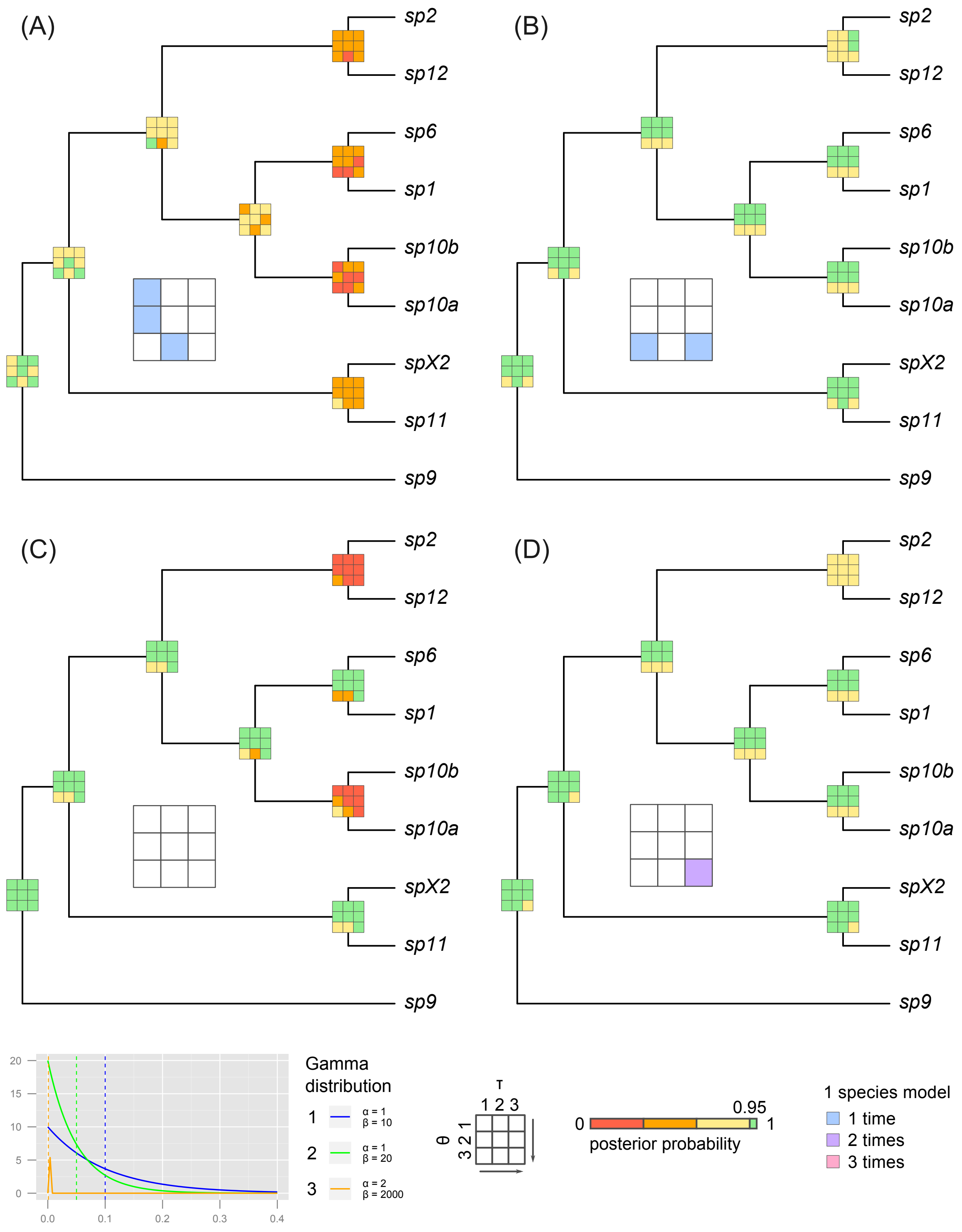
(A)
(B)
(C)
(D)
sp2
sp12
sp6
sp1
sp10b
sp10a
spX2
sp11
sp9
Gamma distribution
1 α = 1 β = 10
2 α = 1 β = 20
3 α = 2 β = 2000
τ
1 2 3
θ
1 2 3
0
0.95
1
posterior probability
1 species model
1 time
2 times
3 times

Figure S8. Overview of the results from the different species delimitation methods and data. Inferred entities that were fully congruent with the *a priori* morphospecies assignments are indicated by the bold circumscribed coloured squares, incongruent units remain white; sub-splitting within morphospecies is indicated by horizontal dashes. Additional sub-splitting within morphospecies that share overlapping MOTUs are circumscribed by a narrow line. Uncertain delimitations are indicated by thin lines between *a priori* morphospecies.

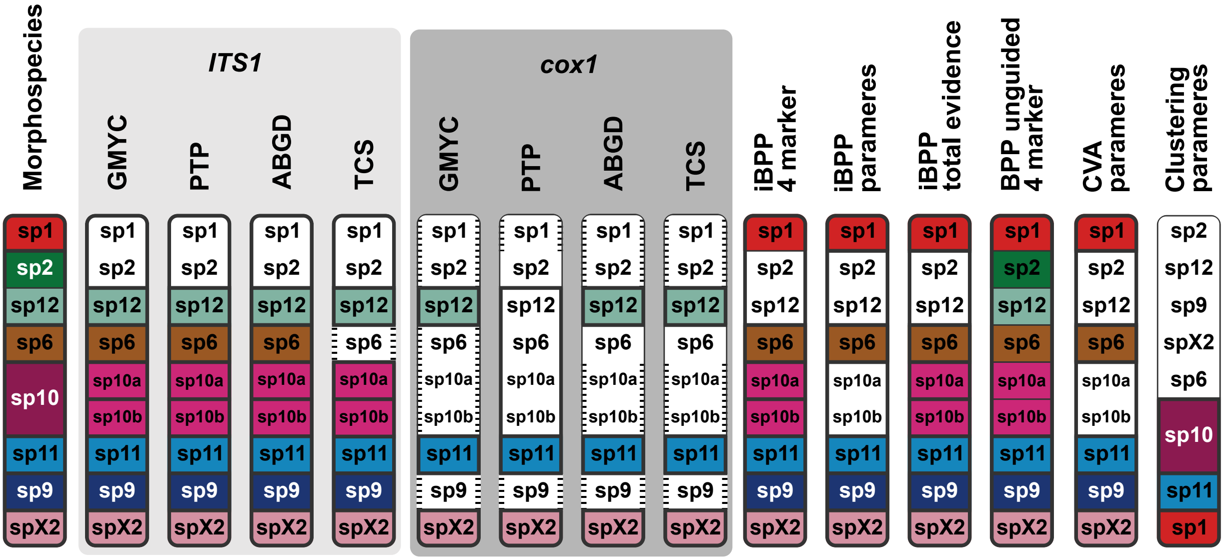

Morphospecies
ITS1
cox1
GMYC
PTP
ABGD
TCS
iBPP 4 marker
iBPP parameres
iBPP total evidence
BPP unguided 4 marker
CVA parameres
Clustering parameres
sp1
sp2
sp12
sp6
sp10
sp10a
sp10b
sp11
sp9
spX2
