# Supplementary figures and images for: Bayesian species delimitation in Pleophylla chafers (Coleoptera) – the importance of prior choice and morphology
Source: BMC Evol Biol. 2016 May 5;16:94. doi: 10.1186/s12862-016-0659-3 (PMC4858874; doi:10.1186/s12862-016-0659-3)

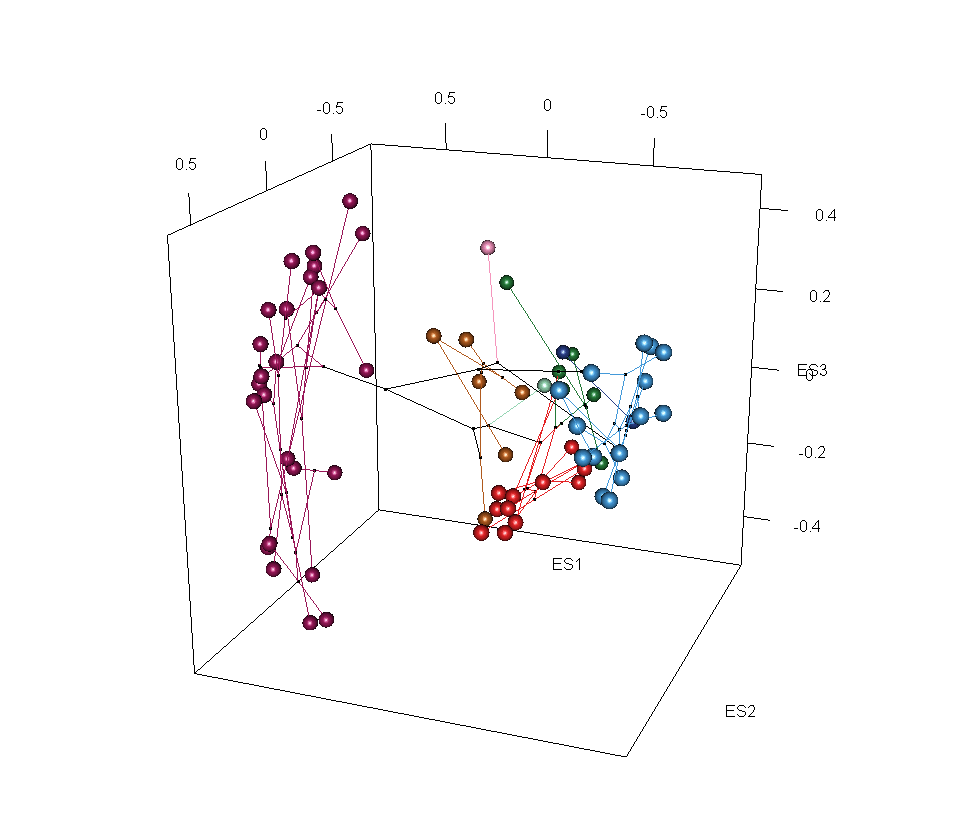

Supplement: Additional file 2: — 3d morphospace. (GIF 6172 kb) [file 12862_2016_659_MOESM2_ESM.gif]
